# Benchmarking Procedures for High-Throughput Context Specific Reconstruction Algorithms

**DOI:** 10.3389/fphys.2015.00410

**Published:** 2016-01-22

**Authors:** Maria P. Pacheco, Thomas Pfau, Thomas Sauter

**Affiliations:** ^1^Systems Biology Group, Life Sciences Research Unit, University of Luxembourg, LuxembourgLuxembourg; ^2^Department of Physics, Institute of Complex Systems and Mathematical Biology, University of AberdeenAberdeen, UK

**Keywords:** metabolic networks and pathways, metabolic reconstruction, constraint-based modeling, tissue specific networks, benchmarking, validation

## Abstract

Recent progress in high-throughput data acquisition has shifted the focus from data generation to processing and understanding of how to integrate collected information. Context specific reconstruction based on generic genome scale models like ReconX or HMR has the potential to become a diagnostic and treatment tool tailored to the analysis of specific individuals. The respective computational algorithms require a high level of predictive power, robustness and sensitivity. Although multiple context specific reconstruction algorithms were published in the last 10 years, only a fraction of them is suitable for model building based on human high-throughput data. Beside other reasons, this might be due to problems arising from the limitation to only one metabolic target function or arbitrary thresholding. This review describes and analyses common validation methods used for testing model building algorithms. Two major methods can be distinguished: consistency testing and comparison based testing. The first is concerned with robustness against noise, e.g., missing data due to the impossibility to distinguish between the signal and the background of non-specific binding of probes in a microarray experiment, and whether distinct sets of input expressed genes corresponding to i.e., different tissues yield distinct models. The latter covers methods comparing sets of functionalities, comparison with existing networks or additional databases. We test those methods on several available algorithms and deduce properties of these algorithms that can be compared with future developments. The set of tests performed, can therefore serve as a benchmarking procedure for future algorithms.

## 1. Introduction

Metabolic network reconstructions become ever more complicated and complete with reconstructions like Recon2 (Thiele et al., 2013) or HMR (Mardinoglu et al., 2014) containing more than 7000 reactions. While these reconstructions are a great tool for the analysis of the potential capabilities of an organism, one challenge faced by many researchers is that different cell types in multicellular organisms exhibit diverse functionality and the global generic network is too flexible. This issue has been addressed in two ways, by manually generating tissue specific models (Gille et al., 2010; Quek et al., 2014) or by creating algorithms for automatic reconstructions (Becker and Palsson, 2008; Jerby et al., 2010; Zur et al., 2010; Agren et al., 2012; Wang et al., 2012; Vlassis et al., 2014; Yizhak et al., 2014; Robaina Estévez and Nikoloski, 2015). Ryu et al. (2015) and Robaina Estévez and Nikoloski (2014) recently reviewed this field and give a good overview of the available reconstructions and point to many algorithms used in this context. While Ryu et al. (2015) are more concerned with the the state of the reconstructions, Robaina Estévez and Nikoloski (2014) focused on the applicability and properties of the available algorithms. With that many methods available, the method selection is difficult, and it is an enormous effort to try and distinguish which network, of a set of generated networks is best. Quality assessment is therefore essential but the methods used to evaluate the currently available algorithms are very diverse and it is difficult to compare them with each other. There are several approaches for validation which can essentially be split into two different categories: Consistency testing and Comparison based testing. The first is concerned with robustness against noise, e.g., missing data, and whether distinct sets of input data yield distinct models. The second commonly aims at validating the resulting model against other models or against additional data. Comparison tends to be the more common approach so far, while consistency is often ignored. This leads to the problem that algorithms are often prone to be over-specific to the comparison dataset (e.g., parameters like expression thresholds or weights working well for only one specific tissue). While comparison methods validate the reconstructed model, they are however not validating the consistency. Thus, it is possible that small differences in the input dataset can lead to vastly different networks, or even very diverse datasets yield the same models. The latter is particularly true if e.g., a biomass function is set as objective function, since it will lead to the inclusion of a multitude of reactions, which might not be necessary if a specific tissue is supplied with some metabolites by other tissues. To investigate the quality of automatically reconstructed networks it is therefore necessary to rigorously test them. In the following paragraphs, we describe multiple methods that were used in the past. Table 1 also gives an overview of these approaches, and details which concept was used for validation of which algorithm.

**Table 1:** **Overview of methods used for validation of automated tissue specific reconstruction algorithms**.

**Method**	**Used by**
Consistency testing	
Cross validation	PRIME, FASTCORE, MBA, FASTCORMICS, iMAT
Diversity of generated models	GIMME, mCADRE, tINIT, FASTCORMICS
Comparison based testing	
Comparison with manually curated network	INIT, MBA
Comparison with additional databases	mCADRE, RegrEx, iMAT
Comparison with shRNA knockdown screens	MBA, FASTCORMICS
Comparison with literature mining	iMAT
Comparison with metabolic exchange rates	PRIME
Comparison with known metabolic functions	MBA, mCADRE, FASTCORE

### 1.1. Methods for testing algorithmic consistency

The idea of consistency testing covers two major aspects: Robustness of the method and its capacity to distinguish slightly different contexts.

If feasible, random cross validation of the resulting models for a given set of input data can help to determine the robustness of the method with respect to noisy data (Vlassis et al., 2014). Left-out cross-validation allows identifying the reactions that if left-out from the input set would nevertheless be included (or excluded for inactive reactions) in the output model as their inclusion is supported by other reactions of the input set (Pacheco et al., 2015). The robustness of algorithms against noise can also be assessed by adding noise to the expression data i.e., by using a weighted combination of real and random data (Machado and Herrgård, 2014). The main issue of using random and left-out cross validation with most of the current algorithms is that running times of several hours makes decent cross-validation with hundreds of test and validation sets infeasible. While small cross validation runs (e.g., when multiple sources of input data are available and only some sets are considered, Jerby et al., 2010) can give an indication of robustness, they cannot replace random sampling runs, which reflect noisy data much better.

To test the diversity of generated networks, many algorithms are employed to generate multiple networks and those networks are then investigated for dissimilarity (Becker and Palsson, 2008; Wang et al., 2012; Agren et al., 2014; Pacheco et al., 2015; Uhlén et al., 2015). If networks of similar cell types group together in a clustering and networks of divergent cell types are further apart, this indicates that the method does indeed generate specific networks. While it is desirable to obtain distinct networks for distinct tissues, the optimal method should not be too sensitive to small changes in the input data. Otherwise the resulting networks are prone to overfitting to the provided input data.

### 1.2. Methods for comparison based testing

Comparison based testing is commonly employed to show the advantages of the presented algorithm compared over previous algorithms or to show the quality of the reconstructed network based on additional, formerly unknown, data. While the former has been employed for the validation of some algorithms (Wang et al., 2012; Vlassis et al., 2014; Robaina Estévez and Nikoloski, 2015), and becomes more important with an increasing number of available methods, it has also recently been used to compare multiple methods systematically (Machado and Herrgård, 2014; Robaina Estévez and Nikoloski, 2014). In the review by Machado and Herrgård (2014) 8 different methodologies (including GIMME, Becker and Palsson, 2008, iMAT, Zur et al., 2010 and a method by Lee et al., 2012) where tested on an independent dataset. However, their focus was on comparing the quality of flux value predictions, i.e., flux bounds specific to a condition in *Escherichia coli* and yeast, and not the reconstruction of tissue specific networks, i.e., the extraction of an active sub-network.

#### 1.2.1. Comparison against manually curated networks

Comparison to a manually curated tissue was employed by Agren et al. (2012) for the INIT algorithm, when they compared their automatically generated liver reconstruction to HepatoNet. However, they were restricted to a comparison on the gene level, since the source network used by INIT was the HMR database (Mardinoglu et al., 2013), while HepatoNet used its own identifiers. As they mention the difference between the reconstructed and manually curated models was partially due to absence of genes from HMR that were present in HepatoNet. Simultaneously, it is likely that the curators of HepatoNet lacked information on some of the genes present in HMR. Thus, to validate a methodology it is necessary for both the “reference” network and the source network to be compatible.

#### 1.2.2. Comparison against additional datasets and databases

Similarly, many methods compare the resulting reconstructions to additional databases that contain tissue localization data (like BRENDA, Schomburg et al., 2013, HPAm Uhlén et al., 2015 or the Gene Expression Omnibus, Barrett et al., 2013), which was performed for multiple reconstruction methods (Shlomi et al., 2008; Wang et al., 2012; Robaina Estévez and Nikoloski, 2015). The common approach is to check for matches of either genes or proteins that the algorithm assigned to the tissue. This validation (and the results) are however highly dependent on whether the reconstruction method aims at creating a consistent network, or whether it allows inconsistent reactions to be part of the reconstruction. The latter will very likely increase the amount of correctly assigned genes, as enzymatic activities that cannot carry flux in the source reconstruction, would otherwise be excluded. In addition, when extracting reactions from a source network, the associated gene-protein reaction relations are commonly not altered. Thus, genes, which are inactive in a specific tissue show up as assigned to the tissue. Removing them however, could potentially be problematic if the tissue does express the removed gene under a specific condition. In this instance the tissue reconstruction would no longer contain information about this fact, and would indicate wrong potentials of the tissue. Another method that could be used as an assessment for predictive quality of an algorithm was performed by Folger et al. (2011) and subsequently by Pacheco et al. (2015). They used gene silencing data from an shRNA screen and compared it with gene essentiality predictions from a flux balance analysis (FBA) screen. The cancer network generated in this work showed an enrichment of essential genes in the genes indicated in the shRNA screen. In Pacheco et al. (2015), the list of essential genes predicted by FASTCORMICS was further compared to essential genes predicted by PRIME, MBA, mCADRE, and GIMME. Likewise bibliographic approaches have been employed to determine the agreement of reactions belonging to a certain subsystem in the reconstructed network and those subsystems being mentioned in connection with the reconstructed tissue in the literature (Shlomi et al., 2008).

To assess the predictive capability of the Model Building Algorithm (MBA), Jerby et al. (2010) used flux data from a study performed in primary rat hepatocytes and compared the ability of the source reconstruction and the generated reconstruction to predict internal fluxes given the exchange fluxes (and vice versa). This allowed them to assess whether the tissue specific network was indeed performing better in estimating the internal fluxes than the generic reconstruction (in this instance Recon1). They could show that indeed the tissue specific network had a better capability to capture the actual fluxes than the generic reconstruction. This concept was also used by Machado and Herrgård (2014) in their assessment of multiple methods for network contextualization. However, while contextualization commonly aims at altering flux bounds, which leads to a good comparability of flux measurements with predictions, tissue specific reconstruction is aiming at determining the network available in a given tissue. This means that bounds from the underlying source reconstruction are used and these are often unsuitable for the tissue of interest. But as shown by Jerby et al. (2010), even the pure network structure alteration can already improve the agreement between network fluxes and measured data, at least on a qualitative level.

A method developed by Shlomi et al. (2009) to compare the resulting network for the effects of inborn errors of metabolism (IEM) is also often used in model quality assessment. The concept is, briefly, to analyse flux ranges of the exchange reactions of the created network and compare them with clinical indications of increased or decreased metabolite levels. This concept has also been used for assessment of Recon2 (Thiele et al., 2013) who investigated a diverse set of IEMs and could show their effect even on the level of a generic reconstruction. Similarly, the authors of PRIME (Yizhak et al., 2014) used experimentally measured uptake and excretion rates and compared them to the secretion rates determined by the models their algorithm generated. While the former approach is commonly used to provide a qualitative assessment of increase or decrease in production potential, the latter results in a quantitative comparison. However, it requires the availability of uptake and secretion rates, which are commonly only available for cell lines and could be largely different in real tissues.

Another common approach to investigate the quality of reconstructions is the comparison with lists of metabolic functions. This approach is both used to validate automated reconstructions (Jerby et al., 2010; Wang et al., 2012) as well as manual reconstructions (Gille et al., 2010). The aim is to establish whether the reconstruction supports the current knowledge of the target tissue (e.g., a liver reconstruction should support the conversion of ammonia to urea), and to show that there are no structural issues in the reconstructed network (e.g., free regeneration of ATP or reductants).

### 1.3. A benchmark for testing tissue specific reconstruction algorithms

In this paper we present a potential benchmark that is using several of the mentioned methodologies to assess the consistency and quality of reconstructed networks and tested it with several of the available algorithms.

There are however multiple obstacles, when defining a benchmark for contextualization algorithms. There is no such thing as a “perfect” measurement, which will always leave us with noisy data to incorporate. Furthermore, we do not yet have a contextualized model that perfectly reflects a given context which could be used as a target model. In addition, the global reconstructions are not yet complete, and will likely never be and finally, there is a wide variety of data that can be used to contextualize models. Thus, to define a benchmark we will address these questions by generating networks which we define as reference networks for out testing.

The actual benchmark is preceded by a characterization of the algorithms, in which the similarity level of the context-specific reconstructions obtained with real and artificial input data is assessed. In the latter test, artificial models of different sizes were built and 50, 60, 70, 80, and 90% of the reactions of these networks were used as input for the tested algorithms. The capacity of the algorithm to distinguish between different models was compared for the different percentages of input data.

In the actual benchmark, the confidence level of the reactions included in the context-specific reconstructions using real data was assessed by matching z-scores obtained by the Barcode method (McCall et al., 2011) that basically indicate the difference in intensity between the measured intensity and the intensity distribution observed in an unexpressed state and through a comparison against the confidence score at the proteomic level of the Human Protein Atlas (Uhlén et al., 2015). In a second comparison, artificial models were built and 50, 60, 70, 80, and 90% of the reactions of these networks were used as input for the tested algorithms and the output models were then compared to the complete input model. The context-specific networks obtained with the real data were also tested for the functionalities established by Gille et al. (2010).

## 2. Materials and methods

### 2.1. Models used for benchmarking

There are currently two competing global reconstructions for humans available: Recon2 (Thiele et al., 2013) and HMR2 (Mardinoglu et al., 2013). To be able to test multiple validation techniques, we needed to select one of those reconstructions as the source network used by the tested algorithms. We decided to employ Recon2, as we used functionalities originating from HepatoNet (Gille et al., 2010), a model based on Recon1 (Duarte et al., 2007) and largely incorporated into Recon2. However, we still had to modify Recon2 to allow the algorithms to fully reconstruct HepatoNet (the procedure can be found in Supplementary File 1). HepatoNet was also adapted to match reactions and metabolites with Recon2. This modified Recon2 was used as source model for all runs.

In addition to HepatoNet as a comparison model for real data, we constructed ten artificial sub-networks from Recon2. Those networks were generated to be approximately equally spaced in a range between 1000 and 3500 reactions. They were generated by randomly removing up to 4500 reactions from our Recon2 version and determining the consistent part of the remaining model. The first model within ±50 reactions of equally spaced points in the interval [1000, 3500] was selected as representative for this point. The models and model sizes can be found in Supplementary File 5.

### 2.2. Characterization of the algorithms

There are many algorithms available for tissue-specific metabolic network reconstructions (see Table 2). In this section we will detail the algorithms used in our study and give reasons, why others were excluded.

**Table 2:** **Algorithms available for tissue specific metabolic network reconstruction**.

**Algorithm**	**Input**	**Publication**
Akesson04	Set of inactive genes	Åkesson et al., 2004
FASTCORE	Set of active reactions	Vlassis et al., 2014
FASTCORMICS	Gene expression data	Pacheco et al., 2015
GIMME	Gene expression data, objective function	Becker and Palsson, 2008
GIM^3^E	Gene expression data, metabolomics data, objective function	Becker and Palsson, 2008
iMAT	Gene expression data	Zur et al., 2010
INIT	Gene expression data and metabolite presence data	Agren et al., 2012
MBA	High, medium and low reaction sets	Jerby et al., 2010
mCADRE	Gene expression data	Wang et al., 2012
PRIME	Growth rates, gene expression data	Yizhak et al., 2014
RegrEx	Gene expression data	Robaina Estévez and Nikoloski, 2015
tINIT	Gene expression data, functions, metabolite presence	Agren et al., 2014

*Most methods can use expression data as input but there are some that need additional inputs*.

In order to test the algorithms with real data, liver models were built by the tested algorithms using as input 22 arrays from different datasets downloaded from the Gene Expression Omnibus (GEO; Edgar et al., 2002) database (Supplementary File 2). The same data was also used for the cross-validation assays.

#### 2.2.1. GIMME (Becker and Palsson, 2008) and iMAT (Zur et al., 2010)

For the benchmarking of the GIMME (Becker and Palsson, 2008) and the iMAT (Zur et al., 2010) algorithms, the implementation provided by the COBRA toolbox (Schellenberger et al., 2011) was used with an expression threshold corresponding to the 75th percentile. The proceedExp option was set to 1 as the data was preprocessed. For GIMME, the biomass objective coefficient was set to 10^−4^.

#### 2.2.2. INIT (Agren et al., 2012)

In the original paper, INIT (Agren et al., 2012) assigns weights to the genes associated to the input model that were computed by dividing the gene expression in the tissue of interest by the average expression across all tissues. As for the first experiment, only liver arrays were available, z-scores obtained by the Barcode discretization method (Zilliox and Irizarry, 2007; McCall et al., 2011), were used as weights (see below).

#### 2.2.3. RegrEx (Robaina Estévez and Nikoloski, 2015)

The RegrEx implementation in the supplementary files of Robaina Estévez and Nikoloski (2015) was used. This algorithm has previously only been used with RNA-seq data and therefore no established discretization method exist for microarray data. In order to allow a comparison with the others methods, the intensity values after frma normalization and the standard variation were directly mapped to the reactions of the model using the Gene-Protein-Reaction rules (GPR). For reactions that are not associated to any gene, the expression and the standard deviation were set to 0 and 1000, respectively.

#### 2.2.4. Akesson (Åkesson et al., 2004)

For this algorithm, the data was normalized with the frma normalization method and then discretized with Barcode. Genes with z-scores below 0 in 90% of the arrays, were considered inactive and the bounds of the associated reactions, taking into account the Gene-Protein-Reaction rules (GPR), were set to 0. FASTCC (Vlassis et al., 2014) was then run to remove reactions that are unable to carry a flux.

#### 2.2.5. FASTCORE z-score

For FASTCORE z-score, the expression data was normalized with the frma method and discretized using Barcode. Barcode uses previous knowledge on the intensity distribution across thousands of arrays to calculate for each probe set of the analysed array the number of standard deviations to the median of the intensity distribution for the same probe set in an unexpressed state. Genes with a z-score above 5 in 90% of arrays are considered as expressed and mapped to the reactions according to the Gene-Protein-Reaction rules (GPR) to obtain a core set that is fed into FASTCORE (Vlassis et al., 2014).

#### 2.2.6. FASTCORMICS (Pacheco et al., 2015)

The expression values were first normalized with frma, converted into z-scores using Barcode (McCall et al., 2011) and further discretized using an expression threshold of 5 z-scores and an unexpression threshold of 0 z-score. Genes with 90% of the arrays above the expression threshold are assigned a score of 1 while those below the unexpression threshold are assigned a score of −1. All other genes are associated with a discretization score of 0. These scores are then mapped onto the model using the Gene-Protein-Reactions rules to obtain lists of core and unexpressed reactions. Unexpressed reactions are excluded from the model.

The FASTCORMICS workflow allows the inclusion of a medium composition, which was not used in the tests, as the aim was to provide the same information to all algorithms. A modified version of FASTCORE is then run that maximizes the inclusion of core reactions while penalizing the entry of non core reactions. Note that transporter reactions are excluded from the core set but are not penalized.

#### 2.2.7. Context-specific reconstruction algorithm that were not tested

PRIME and tINIT were not included in the tests as they require, in addition to expression data, growth rates for PRIME and information on tissue functionalities for tINIT. Determination of growth rates in multicellular organisms is restricted to cell lines or cancerous cells, as most other cell types are finally differentiated and therefore no longer divide. Since growth rates are an essential part of PRIME it was excluded from the tests. While functionalities are available for some metabolically very active tissues (like kidney and liver), they are often not available for others. Since we wanted to test a wide range of potential tissues, we decided not to employ functionalities in our input set. Therefore, tINIT would be reduced to INIT as the remaining functionality is the same. Since we wanted to focus on gene expression data, which is currently the most readily available type of data, we did not add metabolomic information into our screens. GIM^3^E would need this type of information and was therefore not tested. Finally, MBA, Lee and mCADRE took more than 5 days for a single run on 2 cores of our cluster and where therefore not included.

#### 2.2.8. Similarity of the context-specific models and algorithm-related bias

The similarity level between the context-specific models built by the tested algorithms was assessed by computing the Jaccard index between each pair of models. The matrix containing the Jaccard indices was then clustered using Euclidian distance. Further, for each context-specific model, the number of reactions found by only 1, 2 up to all of the methods was computed and represented as a stacked boxplot. The colored areas represent the different models built by the tested algorithms and for each bin the colored area is proportional to the number of shared reactions.

#### 2.2.9. Sensitivity and robustness testing using artifical data

While there are methods that take continuous expression measurements into account (Colijn et al., 2009; Lee et al., 2012, and reviewed in Machado and Herrgård, 2014), other methods require the user to define sets of reactions that are present (FASTCORE, MBA) or perform some form of discretization to determine the presence or absence of a gene or a reaction (Akesson, GIMME, iMAT, FASTCORMICS). The latter types of methods, using some form of presence/absence calls can be more rigorously tested for robustness, as a target model can be used to provide the present and absent genes/reactions.

We also tested these algorithms using the artificially created networks. The test was performed as follows: The potential available information was defined as the sets of reactions present in each submodel and absent from each submodel. Based on this data different percentages of input information (50%, 60%, 70%, 80%, and 90%) were provided to the algorithms. The same random samples were provided to the tested algorithms to allow a further comparison between the algorithms (generating a total of 5000 models for each algorithm). To be able to use reaction data, we modified the implementation of the GIMME algorithm to allow the direct provision of the *ExpressedRxns* and *UnExpressedRxns* fields. The model similarities were assessed by calculating the Jaccard index between each pair of models generated for input sets from different target models. In addition, the internal distances of all models generated for one target model were calculated (a total of 50,000 comparisons per algorithm). Furthermore, the corresponding models for each algorithm and each tested input percentage were compared, to obtain the inter-algorithm distance.

#### 2.2.10. Robustness testing using real data

For the cross-validation, 20% of the reactions were removed from the core set and transferred to the validation set. The number of these reactions that were included in the output model was determined and a hypergeometic test was computed. The process was repeated 100 times randomizing at each iteration the core set to form different validation sets. For algorithms that take continuous data as input, the cross-validation assay was adapted as follows: 20% of the gene-associated reactions were removed from the input set by setting the expression to 0 and the standard deviation to 1000 for RegrEX and the rxnsScores to 0 for INIT. But only reactions considered to be expressed with a high confidence level formed the validation set i.e., for INIT only reaction with z-scores above 5 and with expression value above 10 for RegrEX. For Akesson the validation set was composed of inactive reactions. The results for Akesson have to be taken with care as the validation set is only composed of 4 reactions. This is due to Barcode only indicating very few genes as absent, which led to only about 40 reactions being removed from Recon2.

### 2.3. Benchmarking with real data

#### 2.3.1. Confidence level of the reactions

The z-scores computed by Barcode give the number of standard deviations of a gene expression level above the mean of the same genes in an unexpressed state. The z-scores of the genes were mapped to the reactions of Recon2 (Thiele et al., 2013), HepatoNet (Gille et al., 2010) and to the context-specific models built by the different workflows using the Gene Protein Rules (GPR). In the same way, the confidence levels assigned by the Human Protein Atlas (HPA) to the proteins of the database were mapped to the reactions of the different context-specific models.

#### 2.3.2. Comparison between different tissue models

The ability of the algorithm to capture metabolic variations among tissues was tested using the GSE2361 dataset (Ge et al., 2005) downloaded from Gene Expression Omnibus (GEO) that contains 36 types of normal human tissues. Twenty-one of the 36 tissues matched tissues in the Human Protein Atlas. The confidence levels of the proteins in the different tissues were first matched to the modified version of Recon2 to determine if proteins with high and medium confidence level are ubiquitously expressed or expressed in a more tissue specific manner. Then the confidence levels were matched to the corresponding context-specific models to verify if the variation observed among the tissue context-specific models matched the one observed in the Human Protein Database.

To further access the quality of the reconstructed models, the fraction of reactions of the Recon2 pathways that are active in the output models were computed. The obtained matrix was then clustered in function of the Euclidean distance (see Supplementary Figure 6).

### 2.4. Benchmarking with artificial data

The runs using artificial data, performed for sensitivity and robustness analysis, were also used to provide an additional benchmarking measurement for the algorithms. Sensitivity, specificity and false discovery rate were calculated by comparison of the reconstructed networks with the respective target network. The artificial nature of these networks allowed us a complete knowledge of the actual target thus making these calculations possible.

### 2.5. Network functionality testing

Function testing is commonly achieved, by defining a set of metabolites that are available and can be excreted and requiring other metabolites to be produced/consumed or a reaction to be able to carry flux. The input and output can either be cast into a linear problem by adding importers and exporters or by relaxing the steady state requirement for the imported and exported metabolites. Gille et al. (2010) used the latter definition and we adapted this approach using the following modification of the standard FBA approach:

$$\begin{array}{l} min\;\sum v_{i}^{+}+v_{i}^{-}\\ \;s.t\;b_{l}\leq S^{\prime }*v^{\prime }\leq b_{u}\\ \;0\leq v_{i}^{+}\leq ub_{i}\;\forall i\in internal\;reactions\\ \;0\leq v_{i}^{-}\leq -lb_{i}\;\forall i\in internal\;reactions\\ \;v_{i}^{+}-v_{i}^{-}=0\;\forall i\in exchange\;reactions \end{array}$$

$$with\;S^{\prime }=[S,\;-S]and\;v^{\prime }=\left[\begin{array}{c} v^{+}\\ v^{-} \end{array}\right]$$

$$b_{l,i}=\left\{ \begin{array}{l} -10000\;\forall i\in imported\;metabolites(-/=)\\ -1\;\forall i\in produced\;objectives(+)\\ 1\;\forall i\in consumed\;objectives(-)\\ \text{0 }else \end{array}\right.$$

$$and\;b_{u,i}=\left\{ \begin{array}{l} 10000\;\forall i\in exported\;metabolites(+/=)\\ -1\;\forall i\in produced\;objectives(+)\\ 1\;\forall i\in consumed\;objectives(-)\\ \text{0 }else \end{array}\right.$$

The test is considered to be successful if there is a non zero value for all evaluators when calculating *S*′·*v*′.

### 2.6. Computational resources

Except for RegrEx, all runs using the liver data were performed on two cores of a 2.26Ghz Xeon L5640 processor on the HPC system of the University of Luxembourg (Varrette et al., 2014) to achieve comparable running times. Tissue comparison runs and artificial simulation runs were performed on the same cluster but not limited to specific node types.

## 3. Results

### 3.1. Characterization of the algorithms

#### 3.1.1. Similarity of the context-specific models and algorithm-related bias

The aim of this characterization step is to categorize the algorithms based on the similarity of their output models in order to gain insight into algorithm-related bias, requirements of the algorithms i.e., thresholds and more importantly when to use which algorithms. In an ideal case, one would expect that when fed with the same input data, the different algorithms would produce similar networks. But when comparing the context-specific liver models generated with the different algorithms and HepatoNet, only 530 reactions were found in all networks and 77 reactions of our version of Recon2 were inactive in all context-specific models and HepatoNet. The 530 reactions were found among 54 different subsystems, including reactions belonging to pathways expected in all tissues like i.e., the Krebs cycle, glycolysis/gluconeogenesis, but also pathways that were described to take place mainly in the liver, like i.e., bile acid synthesis (Rosenthal and Glew, 2009; Wang et al., 2012) or some reactions of the vitamin B6 pathway (pyridoxamine kinase, pyridoxamine 5′-phosphate oxidase and pyridoxamine 5′-phosphate oxidase; Merrill et al., 1984). This huge variability is due to workflow-related bias and to different strategies and aims of the algorithms. FASTCORE (Vlassis et al., 2014), expects as input a set of reactions with a high confidence level which are assumed to be active in the context of interest and therefore all core reactions are included in the output model (Table 3). In contrast, FASTCORMICS (Pacheco et al., 2015) only includes a core reaction if it does not require the activation of reactions with low z-scores. The main objective of GIMME (Becker and Palsson, 2008) is to build a model by maximizing a biological function. The input expression data is used to identify, which reactions are not required for the objective and can function therefore be removed from the model due to low expression values (Table 3). iMAT (Zur et al., 2010; Lee et al., 2012) and RegrEx (Robaina Estévez and Nikoloski, 2015) maximize the consistency between the flux and the expression discarding reactions that have high expression values if necessary, which might be problematic if reactions have to be included in the model like i.e., the biomass function. INIT (Agren et al., 2012) uses weighted activity indicators as objective, with those having stronger evidence being weighted higher. Whereas the Akesson's (Åkesson et al., 2004) algorithm aims to eliminate non expressed reactions.

**Table 3:** **Models numerics: Size, number of input reactions with high expression, respectively z-score levels, fractions of input reactions set included in the output models, number of genes-associated reactions in the model and running time**.

**Model**	**Size**	**Input reactions**	**Gene-associated reactions**	**Time in seconds**
GIMME	3513	2441	2087	4458
iMAT	3649	2441	2440	2098
INIT	3913	2020	2787	36,002
RegrEx^*^	3239	1626	2576	64
Akesson	5740	1594	3715	54
FASTCORE z-score	2882	1595	2084	17
FASTCORMICS	2663	1595	1906	112

* *Note that RegrEx was run on a different computer with an Intel(R)Xeon(R)CPU E3 1241-v3 @ 3.50 GHz processor*.

**Figure 1:**
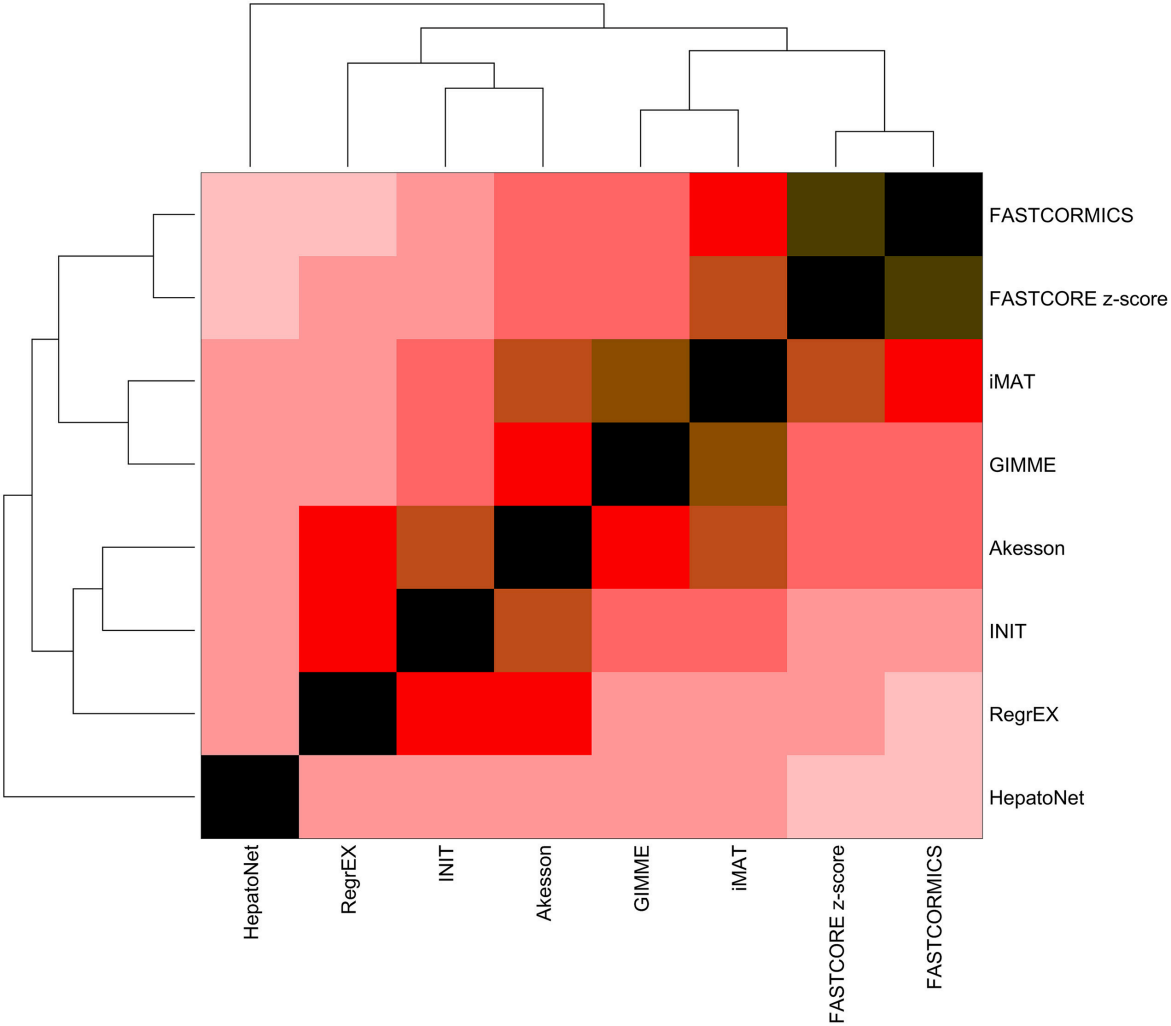
**Similarity index of the models built by the different algorithms**. The Jaccard index was computed for each pair of models and clustered in function of the Euclidean distance. Contrary to what was expected, the output models of the tested algorithms, despite having been fed with the same input show a huge variability.The descritization-based algorithms (GIMME, iMAT, Akesson, FASTCORE, and FASTCORMICS) show the highest similarity levels.

The models, when clustered in function of the Jaccard Similarity Index (Figure 1), form 2 branches and an outlier: HepatoNet. The first cluster is composed of algorithms that take as input continuous data and attempt to maximize the consistency between the data and the Akesson algorithm that eliminates inactive reactions. The second cluster is composed of algorithms that discretize the data in expressed and non-expressed genes. Among this cluster, a second subdivision is observed between the algorithms that used z-score converted data (i.e., FASTCORE z-score and FASTCORMICS) and the ones that use normalized data without further transformation.

Overall the highest similarity level are found between FASTCORE z-score and FASTCORMICS with a score of 85% of similarity followed by iMAT and GIMME with 77% of similarity. The lowest similarity level is found between FASTCORMICS and HepatoNet with only 26% of overlap. The largest overlap between HepatoNet and context-specific reconstructions is found for INIT with 43% of similarity. Note that the INIT model although having as input Barcode discretized data does not cluster with FASTCORE z-score or with the FASTCORMICS models but with RegrEx, suggesting that the choice to consider continuous data rather than defined core set has a larger impact on the output models.

**Figure 2:**
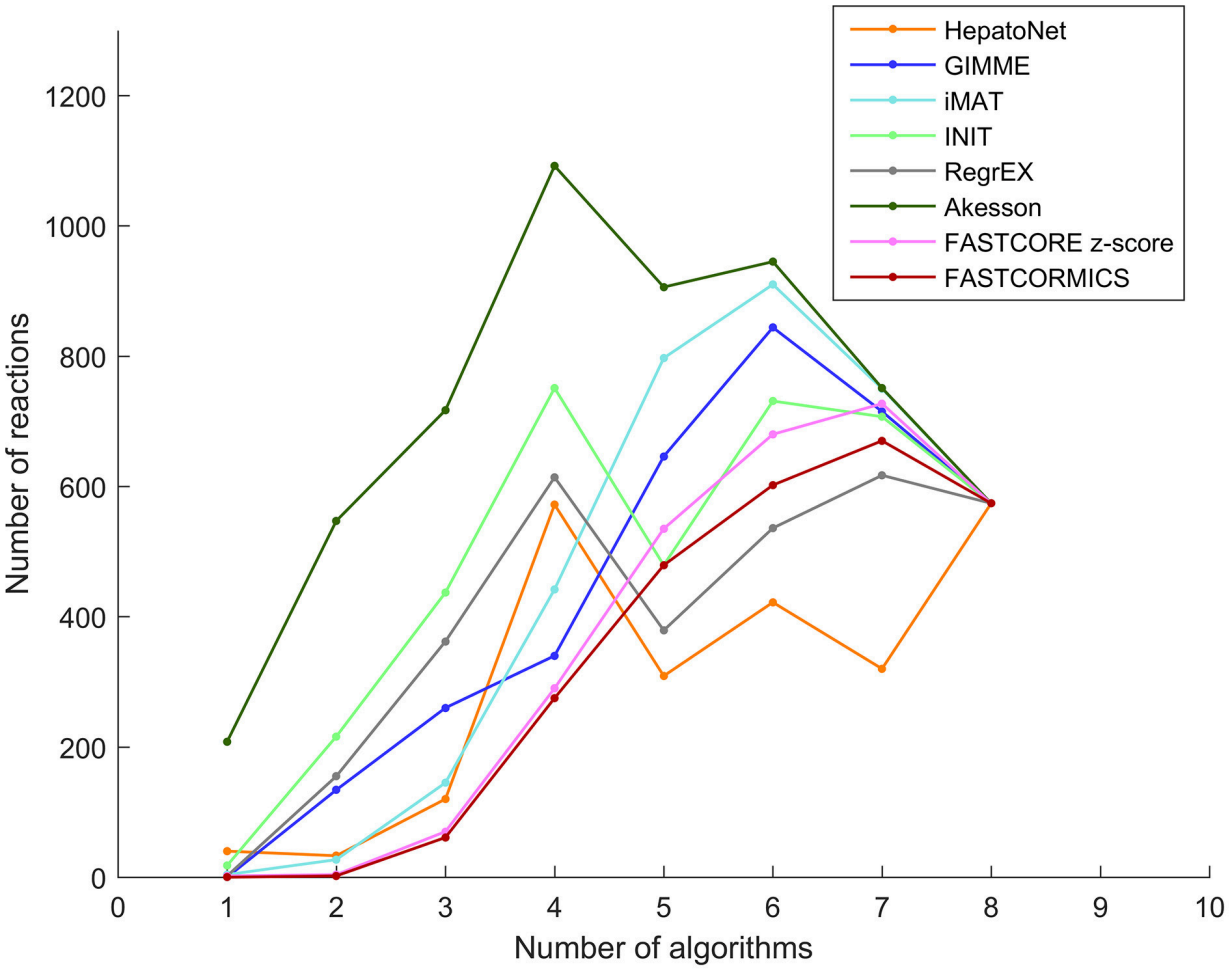
**Reactions overlap: The number of reactions that are shared by the models built by the tested algorithms**. Each line represents HepatoNet or a model built by one of the tested algorithm. The plot illustrates the number of reactions that are common to 1, 2, 3 up to all of the models.

As the algorithms were fed with the same input data, reactions that are predicted by one or only few algorithms are more likely to be algorithm-related bias (Figure 2). The Akesson model that contains 98.56% of the input model includes the largest number of reactions (201) that are absent in the others models.

The reactions included in the FASTCORE, FASTCORMICS, iMAT and GIMME models are for 97%, 98%, 96%, respectively 89% supported by at least 3 other algorithms and display a similar profile shifted to the right. HepatoNet, INIT and the Akesson's model share 92%, 83%, respectively 91% with 3 other algorithms and have different profiles from the algorithms of the first group composed of algorithm that include a discretization step.

In summary, discretization-based algorithms show the highest similarity level and therefore the lowest number of reactions due to potential algorithm-related bias.

#### 3.1.2. Sensitivity and robustness testing using artifical data

Since we noticed that there are two sets of algorithms among the discretizing algorithms, we decided to further test their properties with artificial networks by comparing resulting models from multiple runs for different models and levels of completeness of input data.

**Figure 3:**
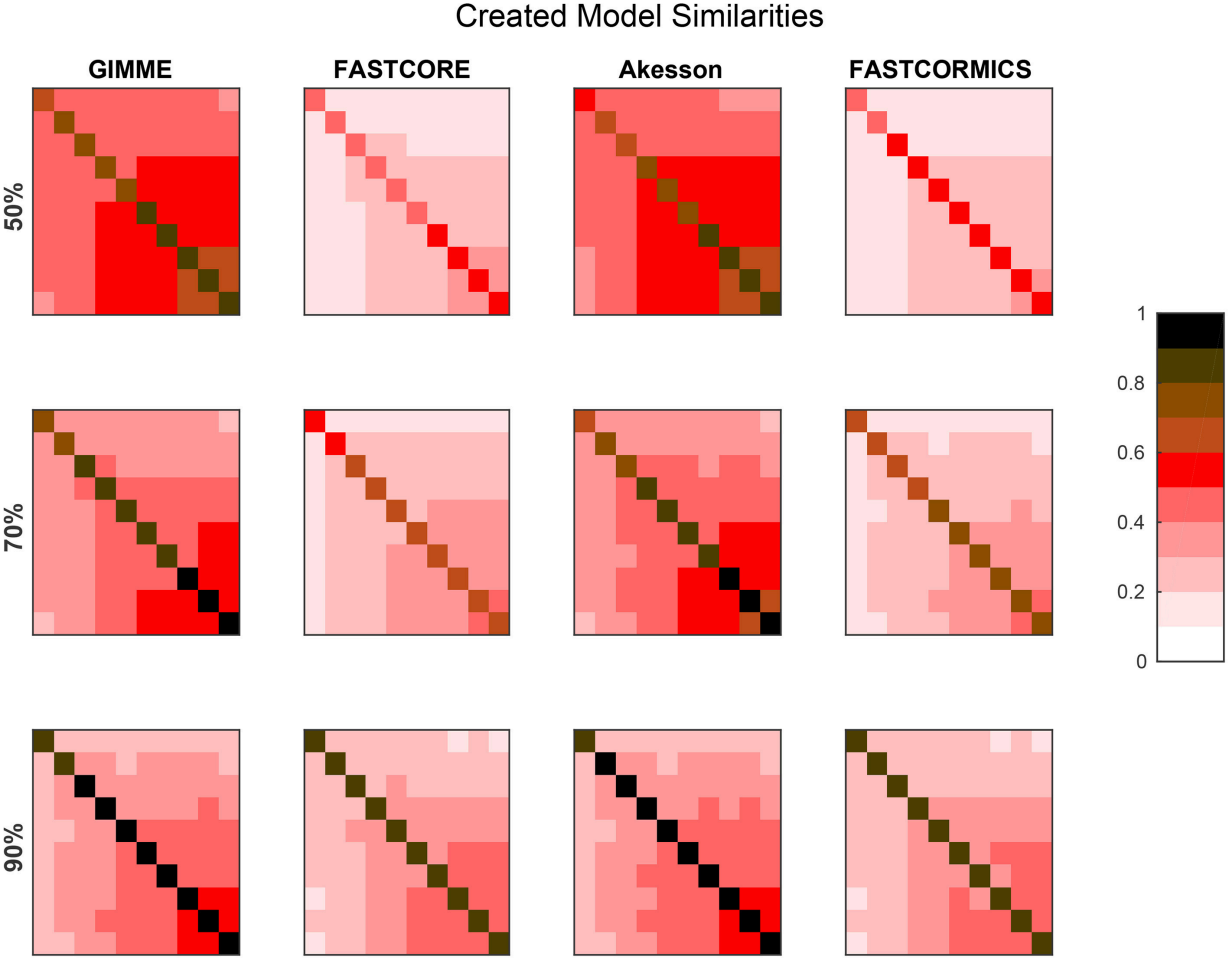
**Resolution power: The plot shows Jaccard distances for the networks generated by the algorithms, when trying to create the artificial networks**. For each of the ten artificial models 100 runs were performed and each square represents the mean Jaccard distance between these networks. E.g., For each percentage and algorithm, the tenth square in the first row is the mean of all pairwise Jaccard distances between the 100 models generated for artificial model 1 (the smallest) and the 100 models generated for artificial model 10 (the largest) generated for the respective algorithm and percentage. The diagonal is the mean of the pairwise Jaccard distances between 100 runs performed. The diagonal can therefore be an indicator for robustness (the brighter, the more similar the models) while the off diagonal indicates similarities between the generated models and is therefore an indicator for specificity to the input (the darker, the more distinct the generated models). When 90% of the data is available, all the algorithms are able to distinguish variations between the different models. But with a less complete data set, inclusive algorithms (here GIMME and Akesson) lose in specificity. It would also be expected that when only 50% of the data is available, the robustness decreases.

**Figure 4:**
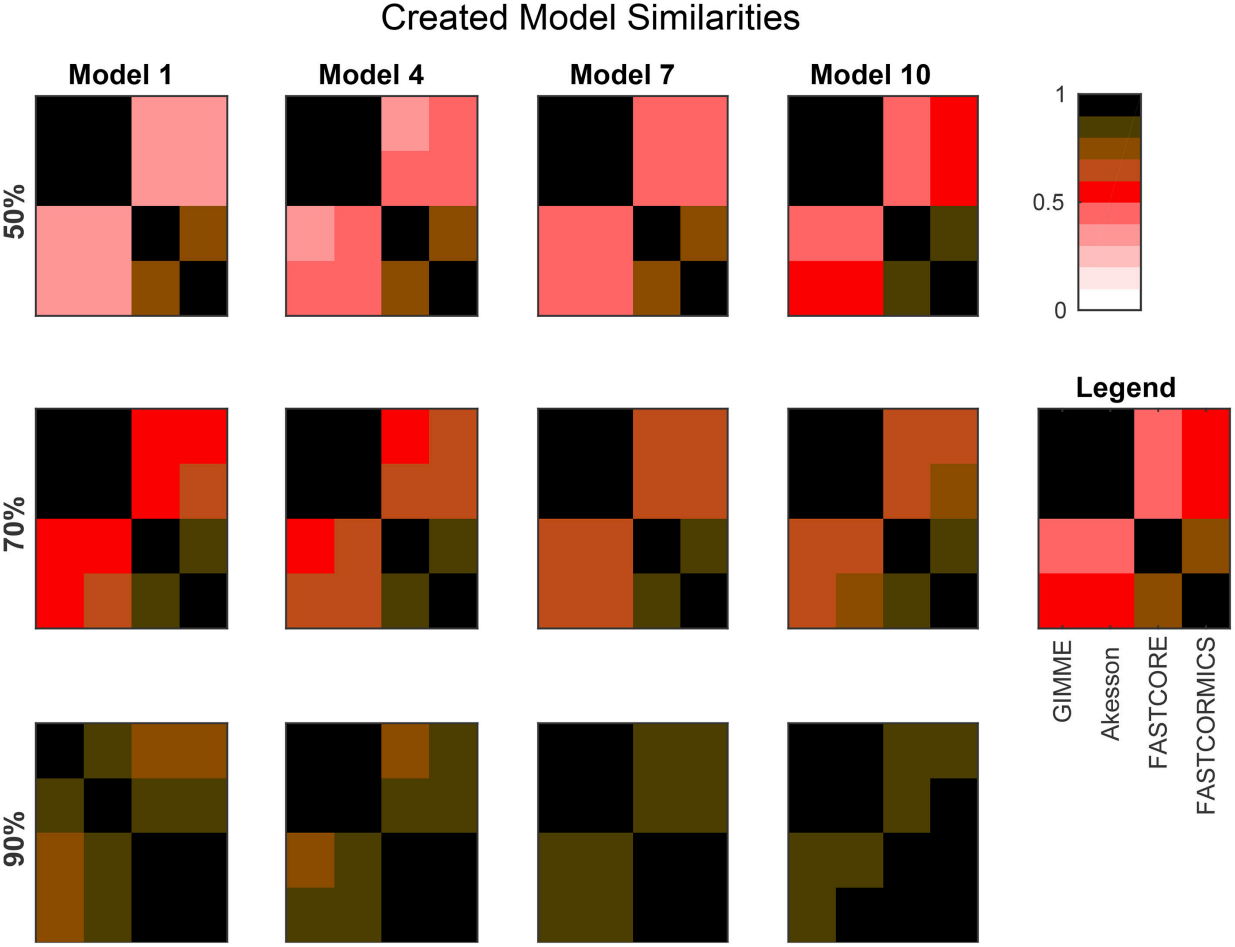
**The plots show the mean Jaccard distance between the networks generated by the different algorithms for several artificial models and input percentages**. For each algorithm, the corresponding networks (using the same input data) are compared. The models are provided in Supplementary File 5. Sizes are: Model 1: 961; Model 4: 1876; Model 7: 2629; Model 10: 3455. Smaller models (e.g., Model 1) tend to yield more distinguishable results, while larger models (due to a larger fraction of common reactions), tend to yield more similar networks. Overall, the difference between inclusive (GIMME/Akesson) and exclusive (Fastcore/FASTCORMICS) algorithms is clearly visible.

Figure 3 provides the average similarities for all models reconstructed for each target model at different available information percentages (A full set of mean similarities for each percentage and each artificial model along with the data for the plots is provided in Supplementary Files 1, 3). Each square represents the mean Jaccard index of the all combinations of networks generated for different input networks [e.g., (1,2) is the average similarity of all networks generated for models 1 to all networks generated for model 2]. The diagonal represents the internal similarity of all networks generated for one model. When 90% of the data is available, all the algorithms are able to distinguish variation between the different models. But with a less complete data set, inclusive algorithms lose in specificity and therefore also progressively lose the capacity to distinguish between different models. Further with 30 and 50% reactions missing, it would be expected that the algorithms get less robust, but Akesson and GIMME only show a modest decrease of robustness (as shown in the diagonal). A similar behavior for the GIMME algorithm was also described by Machado and Herrgård (2014) in a experiment where noise was progressively added to the input data to finally obtain a random input dataset. GIMME showed the same average error in prediction for the random and original data (Machado and Herrgård, 2014), suggesting that due to the optimization of the biomass function, the expression data has a reduced impact on the model building. Comparing the models resulting from runs with different completeness of input data illustrates that the methods tend to converge on more complete data sets, with the Akesson approach and GIMME being more inclusive and the FASTCORE family being more exclusive (see Figure 4 and Supplementary File 4). While initially, with incomplete data, the methods are distinguishable by the networks generated, this difference becomes smaller with additional knowledge.

#### 3.1.3. Robustness testing using real data

In order to further evaluate the confidence level of the reactions included in the different context-specific models a 5-fold cross-validation was performed. The experiment was repeated 100 times with a different validation set. GIMME, iMAT, and FASTCORMICS show the highest robustness, followed by FASTCORE and FASTCORE z-score (see Table 4). Algorithms that maximize the consistency between the data and the flux, e.g., INIT and RegrEx, are less robust with insignificant *p*-value. For Akesson no hyper-geometric test was performed as the validation set was too small to obtain a reliable *p*-value. Note that for context-specific reconstruction algorithms a trade-off has to be found between robustness and the capacity to capture differences between similar contexts. For this reason, a too high robustness might not be desirable as it would imply that the algorithm might lose in resolution power, i.e., the ability to distinguish between different sets of input data. Therefore, it is also advisable to not test for robustness without testing the resolution power.

**Table 4:** **Number and percentage of reactions recovered from the validation set, average model size over 100 reconstruction processes**.

	**Validation set**	**Recovered reactions**	**% of Recovered reactions**	**Sample size**	**Input**	**Hypergeometric *p*-value**
GIMME	488	408 (6.42)	83.57	1878 (6.42)	3871	< 1*e*−100
iMAT	488	335 (10.85)	68.68	1631 (29.85)	3871	< 1*e*−100
INIT	345 (7.16)	83.7	24.26	1931 (113.63)	4469 (7.16)	1
RegrEX	326 (12.79)	160 (19.25)	48.9	2528 (201)	4524 (12.79)	0.96
Akesson	4	0.98 (1.41)	24.5	5343 (6.54)	5828 (24.5)	ND
FASTCORE z-score	319	121.6 (8.26)	38.12	1332 (27.33)	4548	0.0051
FASTORMICS without medium	335(0.4)	192(7.79)	57.14	1516 (27.13)	4782 (7.57)	1e-18

### 3.2. Benchmarking with real data

#### 3.2.1. Confidence level of the reactions included in the different models

**Figure 5:**
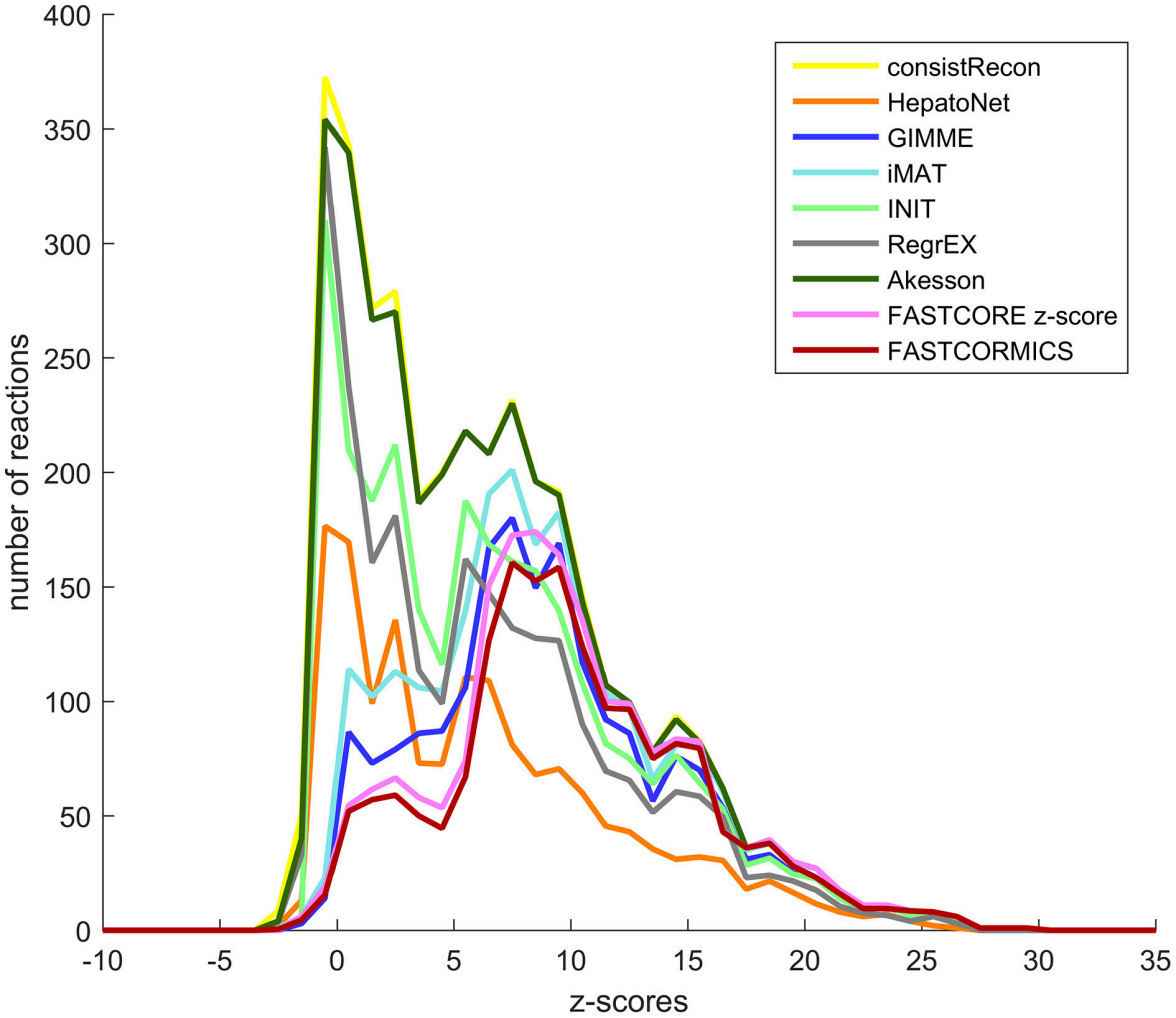
**Confidence score at the transcriptomic level**. Median z-score of the intensity measured in the liver samples to the median intensity distribution for the genes in an unexpressed context mapped the genes-associated reactions of Recon2 (yellow), HepatoNet (orange) the GIMME (dark blue), iMAT (light blue), INIT (green), RegrEx (gray), Akesson (dark green), FASTCORE z-score (pink), and FASTCORMICS (brown) Discretization-based algorithms (GIMME, iMAT, FASTCORE, and FASTCORMICS) are enriched for higher z-score values.

As shown by the previous similarity test, there are several alternative approaches to build context-specific models. To assess the confidence level of a reconstruction, one can quantify the confidence level of the reactions included by each algorithm. Context-specific algorithms assume that the higher the reactions associated expression levels, the more likely the reactions to be active. Following this logic, context-specific reconstructions should be enriched for higher expression levels. As the background level is non negligible and highly dependent on the probes, we corrected for probe effect using the Barcode method. The z-scores computed by Barcode translate the number of standard deviations to the intensity distribution of the same genes in an unexpressed state. The z-scores of the genes mapped to the reactions of Recon2 (Thiele et al., 2013), HepatoNet (Gille et al., 2010) and to the context-specific models built by the different algorithms show that the distribution of the z-scores are for most models shifted, as expected, toward higher z-scores values with a significant *p*-value for all context-specific models except RegrEX (Robaina Estévez and Nikoloski, 2015). Algorithms that use a discretization method show a larger shift to the right than algorithms that maximize the consistency between the flux and the data. Within this group the FASTCORMICS (Pacheco et al., 2015) shows the most significant shift toward the highest z-score values followed by FASTCORE z-score, GIMME (Becker and Palsson, 2008), and iMAT (Zur et al., 2010) (Figure 5 and Table 5). Surprisingly, the consistent version of HepatoNet (Gille et al., 2010) is associated to slightly higher z-scores than Recon2 (Thiele et al., 2013) but significantly lower than most discretization based automated context-specific reconstructions.

**Table 5:** **Comparison between the z-score distribution associated to the models build by the different methods**.

**Model 1**	**Model 2**	**KS *p*-value**
FASTCORE z-score	FASTCORMICS	1e-10
GIMME	FASTCORE z-score	3e-111
iMAT	GIMME	2e-24
INIT	iMAT	< 1*e*−100
HepatoNet	INIT	9 e-18
Akesson	Hepatonet	6e-20
consistRecon	Akesson	0.04
RegRexp	consistRecon	3e-14

*A low p-value indicates that the z-score distribution of Model 2 is shifted toward higher values (to the right) compared to Model 1 (Kolmogorov-Smirnov test)*.

Further, unlike their competitors, all the discretization-based context-specific reconstructions show an enrichment of genes with a high and medium confidence scores to be expressed at the protein level (Uhlén et al., 2015). A stronger enrichment is observed for FASTCORE z-score and FASTCORMICS with 46 and 50% of the gene associated reactions having a high or medium confidence level Table 6, respectively. GIMME and iMAT include 28 and 30% reaction with high or medium confidence levels, respectively. Again surprisingly, HepatoNet does not show an enrichment for high and medium confidence levels.

**Table 6:** **Number, percentage of gene-associated reactions and percentage of reactions of each context-specific reconstruction that have a high, medium and low confidence score to be expressed at the protein level**.

**Algorithms**	**Description**	**High**	**Medium**	**Low**	**Not detected**
	Number of reactions	628	641	65	265
Recon	% of the reactions of the model	11 %	11 %	1 %	5 %
	% of the gene-associated reactions	17 %	17 %	2 %	7 %
	Number of reactions	213	266	47	108
HepatoNet	% of the reactions of the model	9 %	11 %	2 %	5 %
	% of the gene-associated reactions	12 %	15 %	3 %	6 %
	Number of reactions	518	444	47	126
GIMME	% of the reactions of the model	15 %	13 %	1 %	4 %
	% of the gene-associated reactions	25 %	21 %	2 %	6 %
	Number of reactions	574	525	55	153
iMAT	% of the reactions of the model	16 %	14 %	2 %	4 %
	% of the gene-associated reactions	24 %	22 %	2 %	6 %
	Number of reactions	453	499	55	155
iNIT	% of the reactions of the model	12 %	13 %	1 %	4 %
	% of the gene-associated reactions	16 %	18 %	2 %	6 %
	Number of reactions	376	418	41	186
RegrEX	% of the reactions of the model	12 %	13 %	1 %	6 %
	% of the gene-associated reactions	15 %	16 %	2 %	7 %
	Number of reactions	624	637	64	260
Akesson08	% of the reactions of the model	11 %	11 %	1 %	5 %
	% of the gene-associated reactions	17 %	17 %	2 %	7 %
	Number of reactions	584	413	21	123
FASTCORE z-score	% of the reactions of the model	20 %	14 %	1 %	4 %
	% of the gene-associated reactions	28 %	20 %	1 %	6 %
	Number of reactions	570	391	15	73
FASTCORMICS	% of the reactions of the model	21 %	15 %	1 %	3 %
	% of the gene-associated reactions	30 %	21 %	1 %	4 %

*An enrichment in high and medium confidence level is observed for discretization-based algorithms (GIMME, iMAT, FASTCORE z-score and FASTCORMICS)*.

In summary, dicretization-based algorithms include reactions with a higher confidence level at the transcriptomic and proteomic level than their competitors.

#### 3.2.2. Comparison between different tissue models

**Figure 6:**
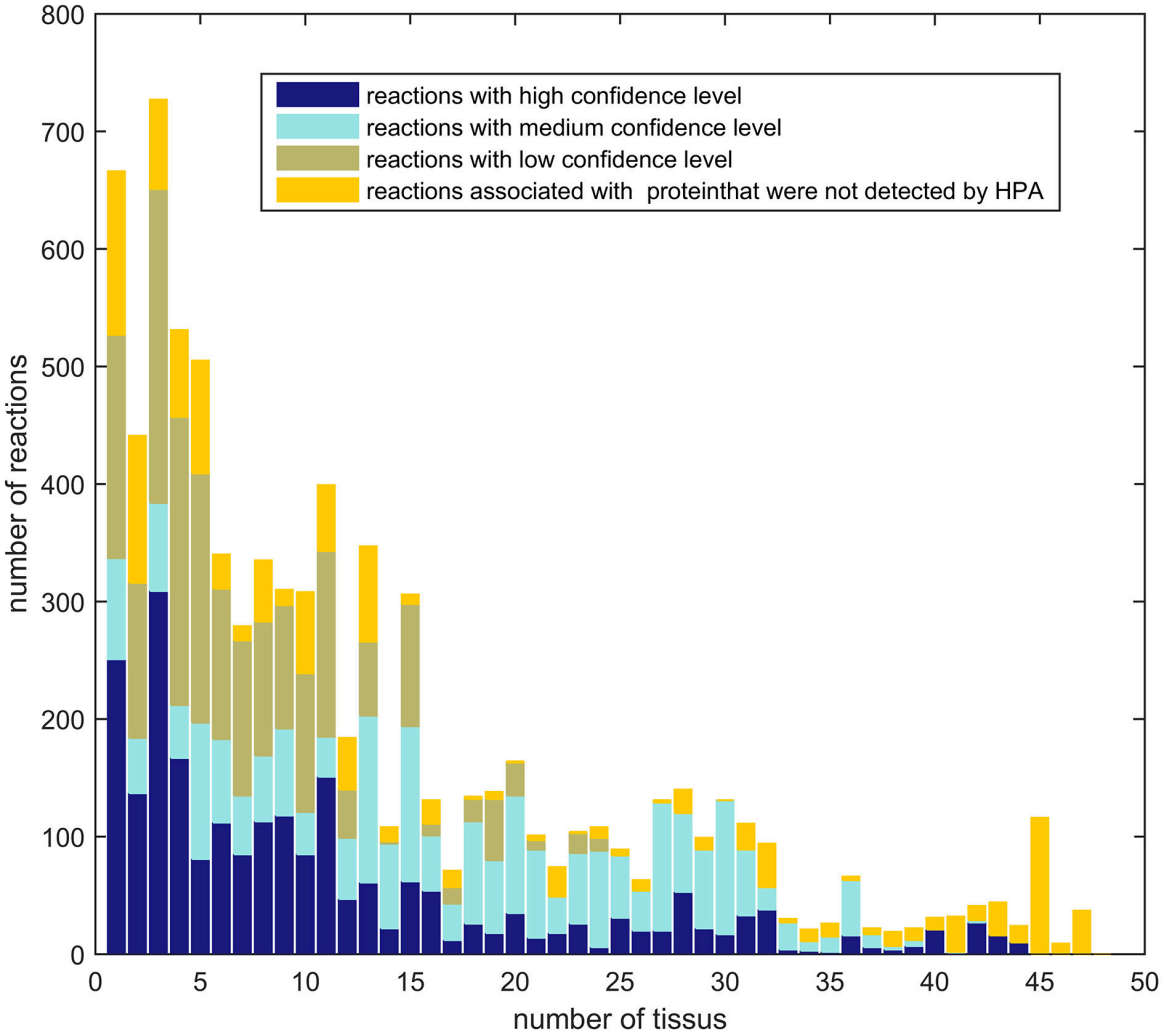
**Ubiquity of expression: Number of reactions of Recon2 with a high or medium confidence level that are shared between 1, 2, 3, up to 48 tissues of the Human Protein Atlas**. Reactions with a high confidence level tend to have a tissue-specific expression.

The aim of a context-specific algorithm, as indicated by the name, is to build models that capture the metabolism of a cell for a given context and therefore these algorithms have to be able to capture variations in the metabolism of different tissues. To pass the following test, context-specific algorithms not only have to be sensitive (or to have a high resolution power) in order capture metabolic difference between tissues, but the reconstructions for different tissues have to be enriched for high or medium confidence levels based on HPA. The last criteria allows to identify algorithms that build different models based on noise or algorithm-related bias. In order to assess the variation among tissues in HPA, the genes with high, medium and low confidence levels for 48 different tissues were mapped to the input model Recon2, showing that very few reactions have a high or medium confidence level in all tissues. In summary, most reactions with high and medium confidence scores have a more tissue-specific expression (Figure 6).

**Figure 7:**
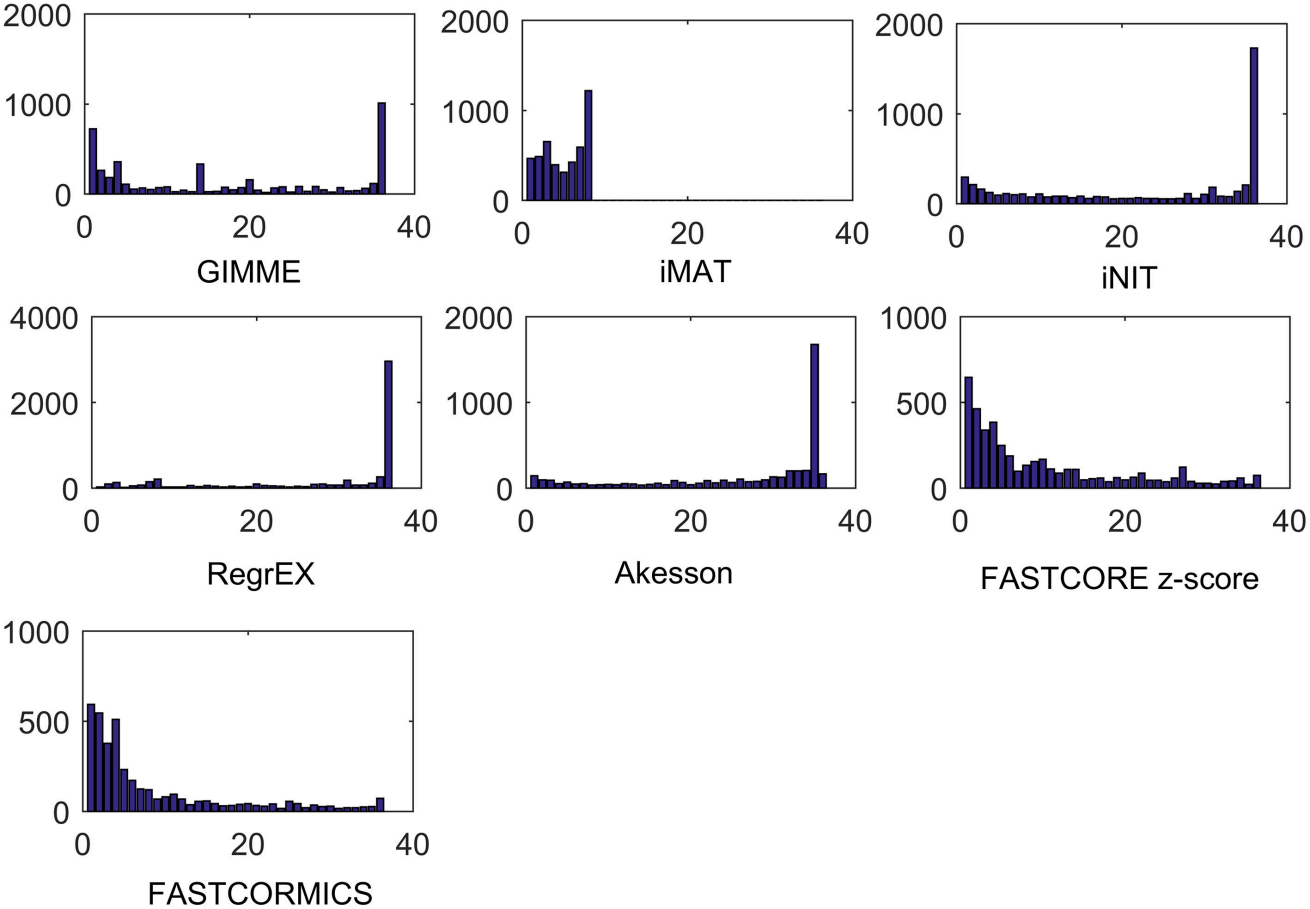
**Tissue specificity of reconstructed models**. Number of reactions that are present in 1, 2, 3, up to 36 tissues models. For INIT and RegrEX, more than 1500 and 3000 reactions are present in all tissues models, while a similar number is present in all but one model created by the Akesson method. Due to computational complexity of iMAT it was only possible to generate 14 out of 36 tissue models.

**Figure 8:**
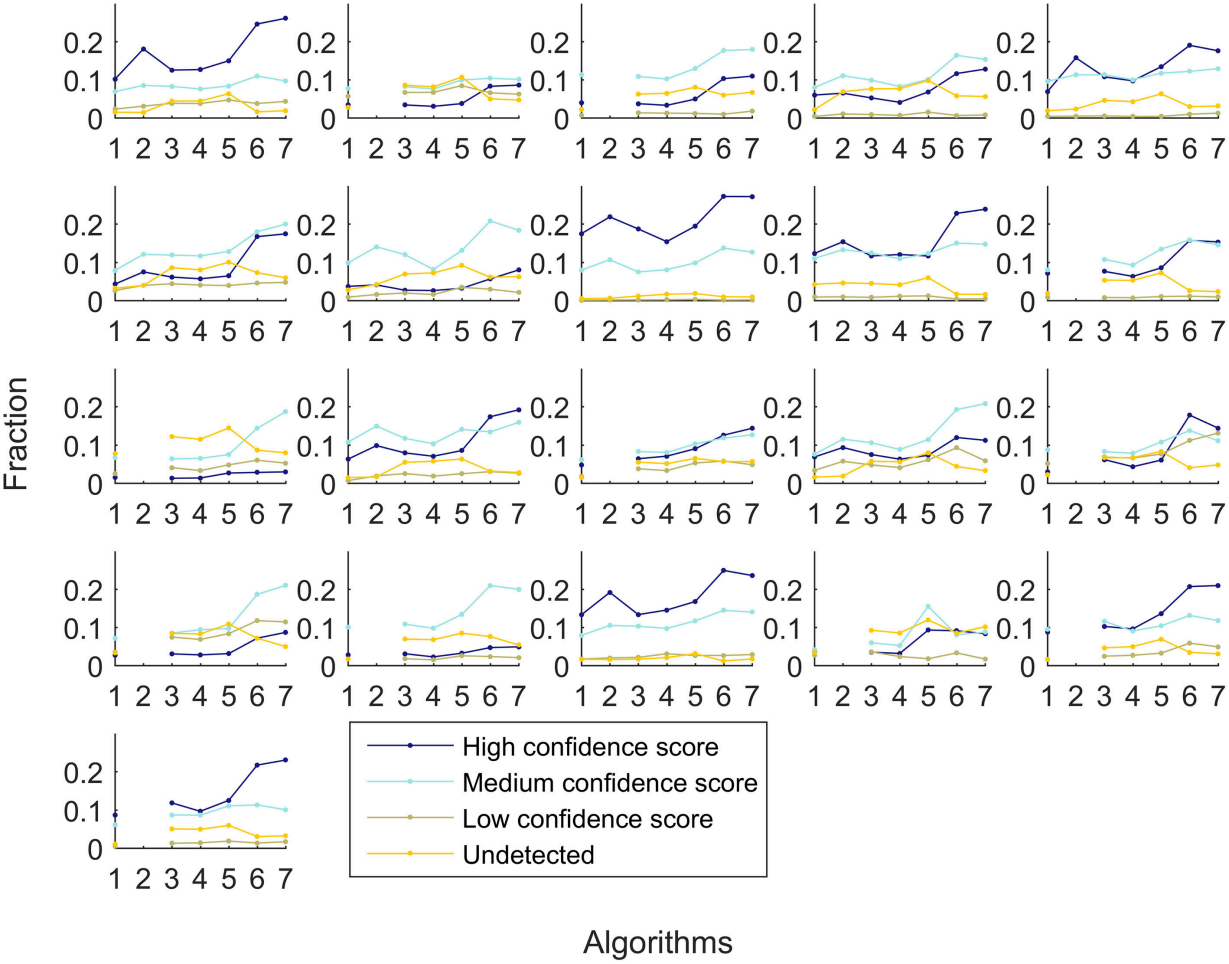
**Fraction of reactions that are associated with high confidence (dark blue), medium confidence (light blue), low confidence HPA level (khaki), and not detected (yellow)**. Each subplot represent a different tissue. The x-axis represent the different algorithms: 1-GIMME, 2-iMAT, 3-INIT, 4-RegrEX, 5-Akesson, 6-FASTCORE z-score, and 7-FASTCORMICS and the y-axis the percentage of reactions.

A similar experiment was performed with context-specific reconstructions built by the tested algorithms, in which the number of algorithms that shared a reactions was assessed (see Figure 7). For RegrEX, INIT and Akesson models, the majority of reactions are found in all tissues. For GIMME, most reactions are either tissue-specific or present in all the tissues. In contrast, the models built by the members of the FASTCORE family show a distribution similar to the that obtained in Figure 6, for HPA. For iMAT only 8 models could be obtained as the computational demands for the reconstructions of the others tissues surpasses the number of core available and the maximal running of 5 days. When looking at the confidence levels associated with the 21 different tissue-specific models, FASTCORE z-score and FASTCORMICS show in 20 out of 21 the highest percentage of reactions with a high or medium confidence level (see Figure 8). The size of the different tissue metabolic models built by the tested algorithm can be found in the Supplementary File 6).

The quality of the tissue-specific models built by the different algorithm were accessed by focusing on selected pathways known to have a more tissue-specific expression, namely bile acid synthesis and heme synthesis. The bile acid synthesis occurs in liver, although one or the other enzyme of the pathways might occasionally be expressed by other tissues (Rosenthal and Glew, 2009; Wang et al., 2012). As expected the FASTCORE family, GIMME and iMAT predicted that the highest fraction of active reactions are found in the liver followed by the foetal liver for the FASTCORE family members and iMAT and by placenta and foetal liver for GIMME. Whereas, the INIT models of skin, bone marrow, corpus, thalamus, pituitary gland and foetal liver had a higher fraction of active reactions than the liver model. Thirteen out of thirty-six of the tested Akesson models predicted 90% and more reactions of the bile acid pathway as active. RegrEX predicted a slightly higher fraction in the thalamus than in the liver and a comparable fraction in the ovary, the foetal brain and the corpus (Supplementary Files 1, 6).

The heme synthesis that occurs mainly in the developing erythrocytes and in the liver (Ajioka et al., 2006), was given as 100% active by the FASTCORE family and completely inactive by GIMME and iMAT in the liver. But these two algorithms predicted the pathway to be active in other tissues. As a matter fact, all the algorithms predicted the pathway to be active in others tissues than the liver. INIT, RegrEX and Akesson included this pathway in 20, 22, and all tested 36 tissues, respectively. Fewer models of the FASTCORE family contained reactions of this pathway: uterus and tyroid for FASTCORMICS and spleen, placenta, uterus, thyroid, skin, bone marrow, amygdala, lung and foetal liver for FASTCORE.

### 3.3. Benchmarking with artificial data

**Figure 9:**
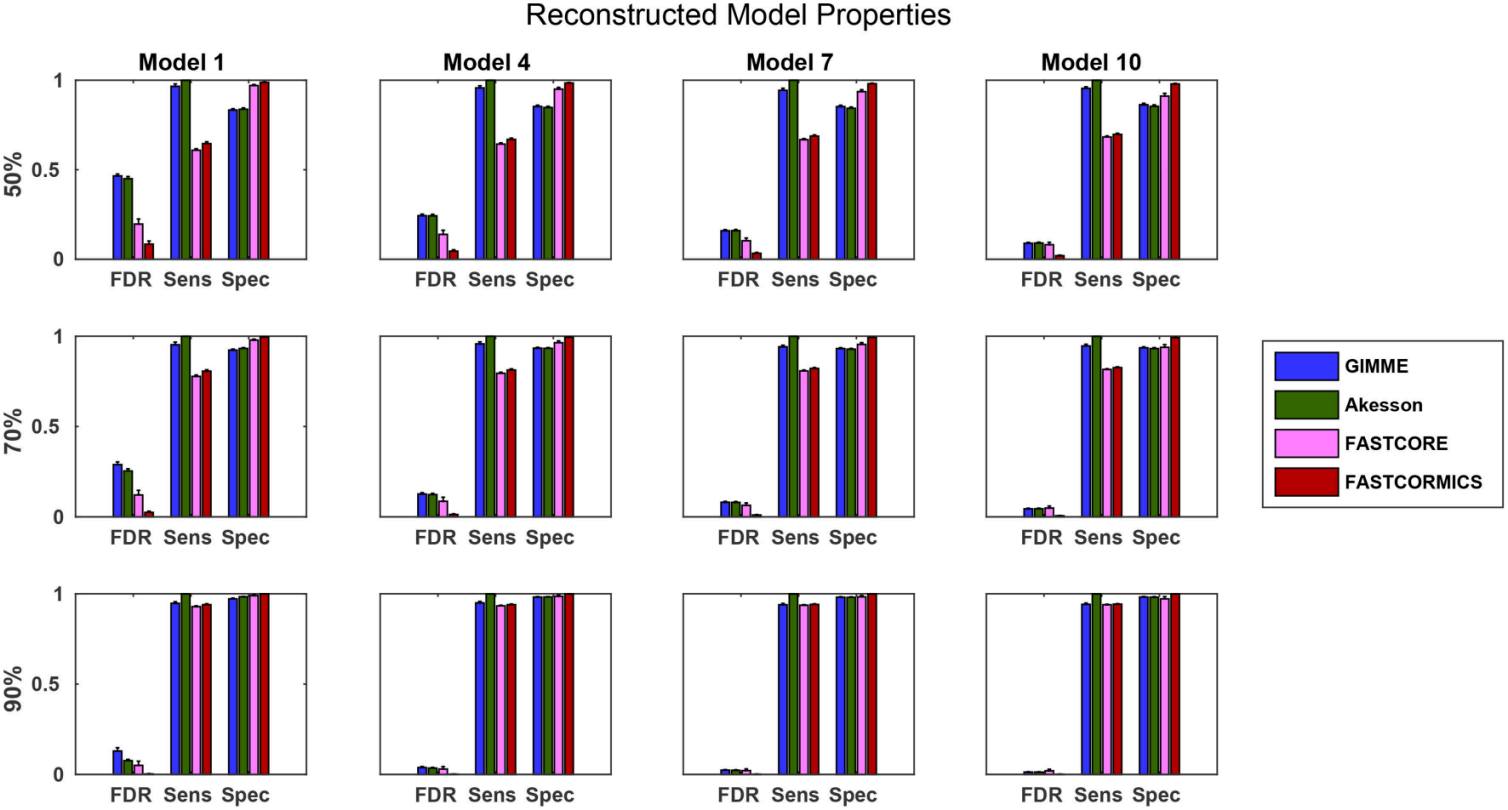
**Statistical measures of the algorithms**. FDR, False discovery rate; Spec, Specificity; Sens, Sensitivity. Data shown is a the mean of 100 runs for each model/input data. The model sizes are: Model 1: 961, Model 4: 1876, Model 7:2629, Model 10: 3455 While the quality of the FASTCORE models is independent of the target model size, the inclusive approaches tend to largely overestimate smaller models, when insufficient data is available. A plot with all models can be found in Supplementary File 1.

To further evaluate the quality of the algorithms, we also used the artificial data (see Section 3.1.2) to benchmark the algorithms. Comparing the resulting models with the target models, we again see that for more complete input sets, the model quality tends to become more similar (see Figure 9). It is interesting to note that the false discovery rate (FDR) of FASTCORE for higher percentages is similar to those of the inclusive models, while FASTCORMICS achieves a better FDR. This indicates alternative routes to activate reactions. In general, there is again the tradeoff between adding too much or too little. It is however interesting that the exclusive algorithms tend to miss targets and their sensitivity is independent on the size of the target model while this is different on inclusive algorithms. Exclusive algorithms show a better FDR than inclusive algorithms. Further, for smaller target models, the loss in precision of inclusive algorithms (1-FDR) is more pronounced for 50 and 70% of the input data, as the inclusive algorithms tend to overestimate the actual model. Similar to the previous experiment, it would be expected that the sensitivity (robustness) would decrease with an increased percentage of missing data. But the inclusive algorithms show an invariant sensitivity in function of the available data suggesting that the expression data has reduced impact on the model building. The specificity for the exclusive algorithms is independent of the target model size and are less affected by the increased missing data than the inclusive algorithms. The sizes of the different reconstructed models also indicates the trend for convergence, and a figure showing the converging sizes is provided in Supplementary File 1.

### 3.4. Functionality testing

**Figure 10:**
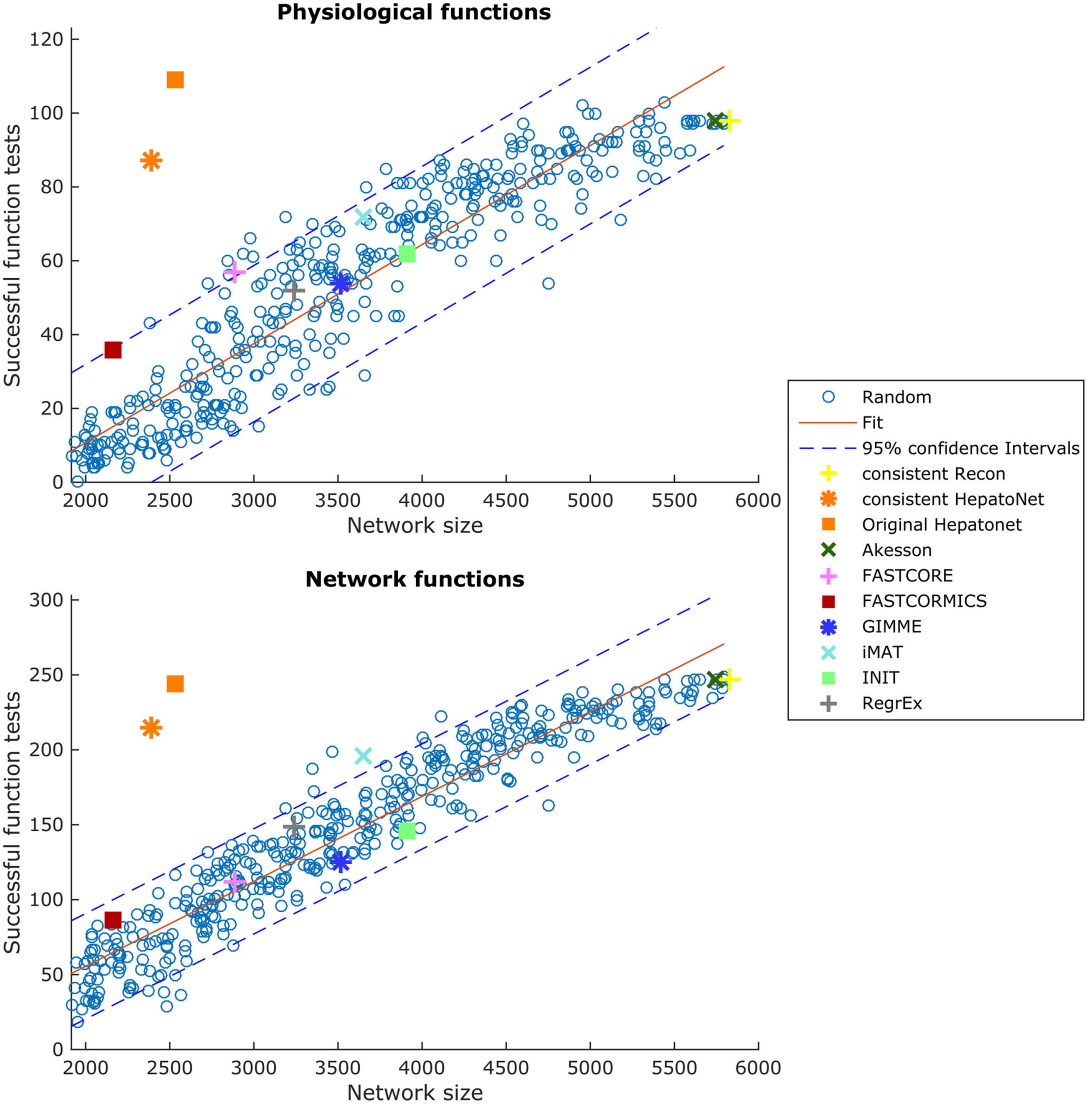
**Scores in the physiological tests correlate with the size of the network**. Two hundred and sixty Random Networks are shown with blue circles.

Functional testing allows us to assess which known functions of a specific tissue are captured by a reconstruction. We used the set of functions defined in HepatoNet and formalized in Section 2.5 for the liver and tested them on all reconstructed networks. We noticed that the success rate of HepatoNet and the generic reconstruction Recon2 are comparable with 244 vs. 247 of 310 network tasks and 109 vs. 98 of 123 physiological tasks for Recon2 and HepatoNet, respectively. The discrepancy with the original publication is likely due to alternative solutions and we noticed that HepatoNet allows free production of NADH and thereby ATP (see Table 2 in Supplementary File 1). The discrepancy between the consistent and inconsistent HepatoNet is due to the formulation of the functionalities, which do not require exchange reactions but modify the b vector, thus generating implicit importers and exporters and allowing inconsistent parts of the network to carry flux. We also noticed an important issue with functional testing: For random models, the larger the models, the higher the functionality score (with *R*^2^ = 0.869 and 0.915 for network and physiological functions, respectively). To illustrate this issue, we generated 400 random networks by removing a random number of up to 2000 reactions from the consistent part of Recon2 and subsequently removing all reactions which could no longer carry any flux. We then tested all network and physiological functions on these networks. The results can be seen in Figure 10, for both the network and physiological tests.

Blue circles represent the random networks; the consistent HepatoNet and the original HepatoNet are displayed in orange, and show a strong enrichment in functionalities. The higher number of functionalities covered in HepatoNet stems from several reactions which are inconsistent, but can be used in a functional testing as described above. We also marked the models generated using the GEO dataset for liver, which score similar to equally sized random models. One of the main reasons for the strong correlation between model size and successful tests is the amount of “positive” testing. Many tests are concerned with some type of biosynthesis or degradation and a larger model is more likely to be able to fulfil these requirements than a smaller model. But even using e.g., the biomass function (like GIMME) as part of the input, the models do not get significantly better than a random model on expression data for liver. None of the algorithms tested achieves high scores in the functionality test and several algorithms are on the lower end of the random network reference. A plot showing the tests passed by the different algorithms is supplied in Supplementary File 7. tINIT could potentially surpass most other algorithms on this test, as it includes functionality information in its reconstruction routine. However, the formulation of tINIT functions is again slightly different from the formulation in HepatoNet and thus not directly compatible.

## 4. Discussion

The primary aim of this work was to review and discuss the existing validation methods and to propose a unified benchmark for the assessment of context-specific reconstruction algorithms. This benchmark will help to identify potential deficiencies of existing and new algorithms and by such increase the quality of context-specific reconstruction algorithms and the models they generate. Although the tested algorithms were validated by their authors in order to be published, the validation methods applied are often incomplete, e.g., a particular aspect of the output model fitting the context of the paper is tested like the ability to produce lactate from glucose in cancer models, leaving other pathways unconsidered. Further, discretization thresholds and other free parameters of the algorithms are likely to be set to optimally fit a particular dataset. Thus, when used in another context the algorithm might perform worse than expected from the original publication. The need of a unified benchmark is nicely illustrated by Figure 1 which shows that despite being fed with the same inputs, the output models vary considerably from each other e.g., the output models of RegExp and FASTCORE that share only around 30% of the reactions.

Part of the variance between the output models is due to different aims and philosophies of the tested algorithms but also due to algorithm-related bias. The second aim of this work was to demonstrate to the users that the context-specific reconstruction algorithms are not equivalent and that the choice of the algorithm and selection of parameter settings for the algorithms have to be performed with care respecting the philosophy of the tested algorithm. For example, GIMME maximizes a chosen biological function and when using GIMME the user assumes that the metabolism of a cell is aimed at the fulfilment of this function. While this biological function can be assumed to be growth for many microorganisms or cancer cells, it is likely to be more complex for multicellular organisms, where multiple “objectives” have to be balanced. In the same way, FASTCORE takes as input core reactions that are always included in the output model and therefore a higher threshold corresponding to a higher confidence level should be set when using FASTCORE.

Although the parameters were set according to the original papers, we are aware that some of the tested algorithms might perform better with a different parameter setting. We decided nevertheless when possible not to change the original parameter settings of the algorithm. First, because the main objective of this paper is not to assess existing algorithms but to propose a benchmark to validate context-specific algorithms. Second the finding of the optimal parameter setting is a computational demanding processes that would require i.e., crossvalidations or other criteria that are not always available. Finding the optimal parameter setting is beyond the scope of a benchmark and rises other questions like overfitting to the data. Third, algorithms should be sufficiently robust to be applied to other datasets with the optimal settings as defined by the authors. As a general principle, in order to avoid overfitting, the parameter estimation should not be performed on the same data than the one used for model generation. We therefore encourage the authors and the users of these algorithms to test them with others parameter settings that might be more appropriate.

The benchmark that we suggest and for which we provide the scripts (http://systemsbiology.uni.lu/software) is based on several criteras: First of all the algorithms have to produce models of high quality that include genes or reactions that are supported by some evidence to be expressed in the context of interest. This aspect was assessed in the workflow by mapping Barcode z-scored gene information and confidence levels established by the Human Protein Atlas to the models. Context-specific reconstruction that extract sub- networks composed only of active reactions in the context of interest from a general reconstruction tend to produce output models that are enriched for genes with high z-scores and a high confidence level to be expressed at the protein level. Indeed although the activity does not correlate perfectly with expression intensities, it was shown that algorithms that exclude reactions with low expression values show a better predictive power than the generic models from which they were extracted. Both tests show that algorithms that perform a discretization of the input data perform better in these tests than algorithms that maximize the consistency between flux values and the data.

We noticed that within the discretizing algorithms, there are two conceptually distinct approaches when considering unsupported reactions. An inclusive concept which considers unknown data as present and an exclusive concept that considers unknown data as absent. Inclusive concepts tend to produce larger networks and score lower, when comparing the networks to additional data, while exclusive concepts tend to produce smaller networks and score higher.

This can be considered as algorithm related bias and it is likely that when multiple algorithms are supplied with the same inputs, reactions that are found by only one or only few algorithms are more likely to be due to algorithm-related bias. Algorithm related bias is not negligible as shown by the huge variability of liver reconstructions with e.g., up to 30% of the reactions being different between the FASTCORE and RegrExp algorithm (Figure 1).

Further, algorithms have to be robust to noise but nevertheless be precise enough to capture the variations in the metabolism of a cell in different contexts i.e., different cell types, different states e.g., healthy vs. disease and eventually between different patients. These two criteria were tested using both experimental and artificial data. Algorithms like GIMME are performing extremely well in the cross-validation assay but score low in the tissue comparison test, as GIMME produces quite similar reconstructions for the different tissues tested. The algorithms using an inclusive concept tend to be more robust to noisy data but have a reduced resolution power. In contrast, exclusive algorithm are less robust as they tend to only recover reactions that are supported by the input data or reactions that are needed to obtain a consistent model, which allow a greater resolution power. Therefore, among the tested algorithms, the FASTCORE family capture best the variation between the different tissues. Further, the confidence level of the reactions included in the 21 tissue models showed that the variability captured by the FASTCORE family models, was not due to noise or algorithm related bias. In the same aspect, the artificial model test gave some interesting insight into the quality of the reconstruction algorithms. While both groups of algorithms, including and excluding, generated about the same model when perfect information was available, they start to diverge at lower amounts of available data. In particular, with less information available the exclusive algorithms underestimate the target network and the including ones overestimate it. While this is to be expected it indicates that the use of two algorithms can give a good approximation of the quality of the available input data and completeness of the reconstruction. If both types of algorithms (inclusive and exclusive) do diverge substantially, it is likely that a relevant amount of input information is missing and that the “true” model is somewhere in between. Similarly, if the models are almost identical, it is likely that the input information and the reconstruction quality is high. GIMME will always include the objective function and all reactions necessary for this function to carry flux. Therefore, those reactions might influence the network size considerably. One advantage of an exclusive concept in this respect, is that its variability is less target model dependent than an inclusive approach. For smaller models, the FDR for inclusive models tends to rise much more rapidly with a more incomplete input data set than for larger models. As we commonly are unaware of the actual size of the target network, this might cause problems when using inclusive approaches.

Another important aspect is the computational demand. To determine the processing time we decided when possible not to change the solver used in the original paper as we noticed that algorithms like e.g., RegrEX are sensitive to the used solver, with gurobi finding an initial solution guess faster than e.g., cplex and thus the result returned by cplex being unusable for the algorithm. The range of computational times is however substantial, with fast algorithms running in seconds to minutes and others taking hours or even days. One of the greatest advantages of faster algorithms, is their capability to be more thoroughly evaluated using cross-validation techniques, which is infeasible for an algorithm running several days. We also observed an issue when running the INIT algorithm. For unknown reasons, the algorithm consistently stopped after 10 h of computation. In particular, the resulting models were odd at best, as they should be close to the models generated by FASTCORE, and in the artificial test, should be optimal on optimal inputs. However, the artificial test was far from optimal, and we assume that the solver does terminate computation at some point.

Finally, we also assessed the capacity of the context-specific reconstruction to pass the functional test as established in Gille et al. (2010). We found that no algorithm outperforms random models, but that a fitted model can indeed show higher scores without adding more reactions, as seen in Figure 10. Unfortunately, obtaining functional data is a very time consuming process and necessitates intensive literature research every time a new tissue model is created. The failure of the tested algorithms in the functional test is mainly due to the high number of non-gene associated reactions in the generic input model (one third of Recon2) and due to the reactions associated to genes with low expression levels. The tested algorithms extract a sub-network from the input model that includes all or most reactions associated with high expressions levels (core) and few reactions with low expression levels (non-core) in order to obtain a consistent model. A slightly different core reactions set, can cause the core reactions to be connected in a different way and as a result the model displays different functionalities. As the choice of the non-core reactions is to a large extent not guided by the data, the obtained functions are random as shown by the functionality test. Interestingly, the reactions found in HepatoNet do have weak evidence when compared to HPA or z-scores, which partially provides another explanation for the inability of the tested algorithms to recover these activities. This however indicates that the general reconstruction currently used lacks either the correct gene-protein-reaction associations for several reactions necessary for the functionalities in liver, that there are alternative pathways missing in the reconstruction and the reactions used in HepatoNet are not the “true” reactions, that the functions are incorrectly assumed to be available in liver or that the functionality lacks information about the consumed cofactors. Indeed, as all the exchange reactions are closed, some reactions might not carry a flux as the associated cofactor cannot be regenerated. This would also explain why bigger models accumulate more functions. The larger the models, the higher the likelihood of internal loops that could allow a regeneration of cofactors. Further it might also indicate that transcriptomics alone might not be sufficient to build functionally correct models. Information on the uptake and excreted metabolite added to the input reactions set would probably increase the score of most algorithms. We did nevertheless not include this type of information in the input data as the latter is not available for *in vivo* tissues. While presence of importers and exporters does not influence the functional tests, they are however highly influenced by the availability of internal transporters.

Assuming that the defined functions are indeed present in liver, this would indicate the importance of algorithms like tINIT which do take these functionalities into account and which could, given the right reference network, indicate potential missing links in the current reconstructions. tINIT is nevertheless not able to capture metabolic differences between different tissue as shown in Uhlén et al. (2015), calling for a new generation of algorithms that capture metabolic variation and that are able to take as input functionalities. Note here that algorithms like PRIME that do not extract a subnetwork to obtain a context-specific model, but modifies the bounds of the reactions of the input model, will have regardless of the modeled cell-type or context the same functionalities as the input model. Therefore, PRIME would score as high as the generic Recon2 in a qualitative test. Nevertheless, the approach used by PRIME is extremely dependant on the accuracy of the growth measurement and biomass formulation, leading to a very variable quality of the flux prediction (Yikzah et al, 2014). In a quantitative test aiming to predict the production rate of lactate by cancer cells, PRIME showed a lower correlation to the experimental data than FASTCORMICS (Pacheco et al., 2015). This suggests that building context-specific algorithms with the discretization-based algorithms and then constraining the uptakes rates of several key amino-acids and glucose as performed in Pacheco et al. (2015) seems to be favorable. Further, as discussed in the main text, there is no unique function to which the metabolism of a non-cancerous pluricellular cell could be reduced and sofar is limited to handle one metabolic function.

In general, we would recommend to assess the quality of an algorithm based on a combination of functional tests for a reconstructed tissue always in comparison to random networks, confirmation using an independent source of information (e.g., proteomics data, when only using expression data for the reconstruction), and an assessment of algorithmic properties, like dependence on target or input model size and dependence on input data quality. For the latter we would suggest using artificial networks to provide a complete knowledge on the expected outcome.

## Author contributions

MP, TP, and TS designed the study. MP and TP implemented the validation methods and performed the calculations. MP, TP, and TS wrote the manuscript.

### Conflict of interest statement

The authors declare that the research was conducted in the absence of any commercial or financial relationships that could be construed as a potential conflict of interest.

## Supplementary material

Supplementary File 1 **Supplementary tables and figures**. Modifications of Recon to allow the reconstruction of hepatonet. Example of free NADPH production in hepatonet.

Supplementary File 2 **List of 22 microarrays used to build the liver models**.

Supplementary File 3 **Data underlying the similarity plots of Figure 3**.

Supplementary File 4 **Data underlying the similarity plots of Figure 4**.

Supplementary File 5 **Artificial Models and used Recon and HepatoNet Models in SBML format**.

Supplementary File 6 **The sizes of the models of the different tissues built by the tested algorithms and the faction of active reactions in each pathway for the different tissues**.

Supplementary File 7 **Successes of the models in the functional tests based on the tests from HepatoNet**.

## References

[B1] Agren R. Bordel S. Mardinoglu A. Pornputtapong N. Nookaew I. Nielsen J. (2012). Reconstruction of genome-scale active metabolic networks for 69 human cell types and 16 cancer types using init. PLoS Comput. Biol. 8:e1002518. 10.1371/journal.pcbi.1002518 22615553 PMC3355067

[B2] Agren R. Mardinoglu A. Asplund A. Kampf C. Uhlen M. Nielsen J. (2014). Identification of anticancer drugs for hepatocellular carcinoma through personalized genome-scale metabolic modeling. Mol. Syst. Biol. 10:721. 10.1002/msb.145122 24646661 PMC4017677

[B3] Ajioka R. S. Phillips J. D. Kushner J. P. (2006). Biosynthesis of heme in mammals. Biochim. Biophys, Acta Mol. Cell Res. 1763, 723–736. 10.1016/j.bbamcr.2006.05.005 16839620

[B4] Åkesson M. Förster J. Nielsen J. (2004). Integration of gene expression data into genome-scale metabolic models. Metab. Eng. 6, 285–293. 10.1016/j.ymben.2003.12.002 15491858

[B5] Barrett T. Wilhite S. E. Ledoux P. Evangelista C. Kim I. F. Tomashevsky M.. (2013). Ncbi geo: archive for functional genomics data sets–update. Nucleic Acids Res. 41, D991–D995. 10.1093/nar/gks1193 23193258 PMC3531084

[B6] Becker S. A. Palsson B. Ø. (2008). Context-specific metabolic networks are consistent with experiments. PLoS Comput. Biol. 4:e1000082. 10.1371/journal.pcbi.1000082 18483554 PMC2366062

[B7] Colijn C. Brandes A. Zucker J. Lun D. S. Weiner B. Farhat M. R.. (2009). Interpreting expression data with metabolic flux models: predicting *Mycobacterium tuberculosis* mycolic acid production. PLoS Comput. Biol. 5:e1000489. 10.1371/journal.pcbi.1000489 19714220 PMC2726785

[B8] Duarte N. C. Becker S. A. Jamshidi N. Thiele I. Mo M. L. Vo T. D.. (2007). Global reconstruction of the human metabolic network based on genomic and bibliomic data. Proc. Natl. Acad. Sci. U.S.A. 104, 1777–1782. 10.1073/pnas.0610772104 17267599 PMC1794290

[B9] Edgar R. Domrachev M. Lash A. E. (2002). Gene expression omnibus: ncbi gene expression and hybridization array data repository. Nucleic Acids Res. 30, 207–210. 10.1093/nar/30.1.207 11752295 PMC99122

[B10] Folger O. Jerby L. Frezza C. Gottlieb E. Ruppin E. Shlomi T. (2011). Predicting selective drug targets in cancer through metabolic networks. Mol. Syst. Biol. 7:501. 10.1038/msb.2011.35 21694718 PMC3159974

[B11] Ge X. Yamamoto S. Tsutsumi S. Midorikawa Y. Ihara S. Wang S. M.. (2005). Interpreting expression profiles of cancers by genome-wide survey of breadth of expression in normal tissues. Genomics 86, 127–141. 10.1016/j.ygeno.2005.04.008 15950434

[B12] Gille C. Bölling C. Hoppe A. Bulik S. Hoffmann S. Hübner K.. (2010). Hepatonet1: a comprehensive metabolic reconstruction of the human hepatocyte for the analysis of liver physiology. Mol. Syst. Biol. 6, 411. 10.1038/msb.2010.62 20823849 PMC2964118

[B13] Jerby L. Shlomi T. Ruppin E. (2010). Computational reconstruction of tissue-specific metabolic models: application to human liver metabolism. Mol. Syst. Biol. 6:401. 10.1038/msb.2010.56 20823844 PMC2964116

[B14] Lee D. Smallbone K. Dunn W. B. Murabito E. Winder C. L. Kell D. B.. (2012). Improving metabolic flux predictions using absolute gene expression data. BMC Syst. Biol. 6:73. 10.1186/1752-0509-6-73 22713172 PMC3477026

[B15] Machado D. Herrgård M. (2014). Systematic evaluation of methods for integration of transcriptomic data into constraint-based models of metabolism. PLoS Comput. Biol. 10:e1003580. 10.1371/journal.pcbi.1003580 24762745 PMC3998872

[B16] Mardinoglu A. Agren R. Kampf C. Asplund A. Nookaew I. Jacobson P.. (2013). Integration of clinical data with a genome-scale metabolic model of the human adipocyte. Mol. Syst. Biol. 9, 649. 10.1038/msb.2013.5 23511207 PMC3619940

[B17] Mardinoglu A. Agren R. Kampf C. Asplund A. Uhlen M. Nielsen J. (2014). Genome-scale metabolic modelling of hepatocytes reveals serine deficiency in patients with non-alcoholic fatty liver disease. Nat. Commun. 5, 3083. 10.1038/ncomms4083 24419221

[B18] McCall M. N. Uppal K. Jaffee H. A. Zilliox M. J. Irizarry R. A. (2011). The gene expression barcode: leveraging public data repositories to begin cataloging the human and murine transcriptomes. Nucleic Acids Res. 39(Suppl. 1), D1011–D1015. 10.1093/nar/gkq1259 21177656 PMC3013751

[B19] Merrill A. H. Jr Henderson J. M. Wang E. McDonald B. W. Millikan W. J. (1984). Metabolism of vitamin b-6 by human liver. J. Nutr. 114, 1664–1674. 6088736 10.1093/jn/114.9.1664

[B20] Pacheco M. P. John E. Kaoma T. Heinäniemi M. Nicot N. Vallar L.. (2015). Integrated metabolic modelling reveals cell-type specific epigenetic control points of the macrophage metabolic network. BMC Genomics 16:809. 10.1186/s12864-015-1984-4 26480823 PMC4617894

[B21] Quek L.-E. Dietmair S. Hanscho M. Martínez V. S. Borth N. Nielsen L. K. (2014). Reducing recon 2 for steady-state flux analysis of hek cell culture. J. Biotechnol. 184, 172–178. 10.1016/j.jbiotec.2014.05.021 24907410

[B22] Robaina Estévez S. Nikoloski Z. (2014). Generalized framework for context-specific metabolic model extraction methods. Front. Plant Sci. 5:491. 10.3389/fpls.2014.00491 25285097 PMC4168813

[B23] Robaina Estévez S. Nikoloski Z. (2015). Context-specific metabolic model extraction based on regularized least squares optimization. PLoS ONE 10:e0131875. 10.1371/journal.pone.0131875 26158726 PMC4497637

[B24] Rosenthal M. Glew R. (2009). Medical Biochemistry: Human Metabolism in Health and Disease. Hoboken, NJ: John Wiley & Sons.

[B25] Ryu J. Y. Kim H. U. Lee S. Y. (2015). Reconstruction of genome-scale human metabolic models using omics data. Integr. Biol. 7, 859–868. 10.1039/c5ib00002e 25730289

[B26] Schellenberger J. Que R. Fleming R. M. T. Thiele I. Orth J. D. Feist A. M.. (2011). Quantitative prediction of cellular metabolism with constraint-based models: the COBRA Toolbox v2.0. Nat. Protoc. 6, 1290–1307. 10.1038/nprot.2011.308 21886097 PMC3319681

[B27] Schomburg I. Chang A. Placzek S. Söhngen C. Rother M. Lang M.. (2013). Brenda in 2013: integrated reactions, kinetic data, enzyme function data, improved disease classification: new options and contents in brenda. Nucleic Acids Res. 41, D764–D772. 10.1093/nar/gks1049 23203881 PMC3531171

[B28] Shlomi T. Cabili M. N. Herrgård M. J. Palsson B. Ø. Ruppin E. (2008). Network-based prediction of human tissue-specific metabolism. Nat. Biotechnol. 26, 1003–1010. 10.1038/nbt.1487 18711341

[B29] Shlomi T. Cabili M. N. Ruppin E. (2009). Predicting metabolic biomarkers of human inborn errors of metabolism. Mol. Syst. Biol. 5, 263. 10.1038/msb.2009.22 19401675 PMC2683725

[B30] Thiele I. Swainston N. Fleming R. M. T. Hoppe A. Sahoo S. Aurich M. K.. (2013). A community-driven global reconstruction of human metabolism. Nat. Biotechnol. 31, 419–425. 10.1038/nbt.2488 23455439 PMC3856361

[B31] Uhlén M. Fagerberg L. Hallström B. M. Lindskog C. Oksvold P. Mardinoglu A.. (2015). Proteomics. tissue-based map of the human proteome. Science 347:1260419. 10.1126/science.1260419 25613900

[B32] Varrette S. Bouvry P. Cartiaux H. Georgatos F. (2014). Management of an academic hpc cluster: the ul experience, in Proceedings of the 2014 International Conference on High Performance Computing & Simulation (HPCS 2014) (Bologna: IEEE).

[B33] Vlassis N. Pires Pacheco M. Sauter T. (2014). Fast reconstruction of compact context-specific metabolic network models. PLoS Comput. Biol. 10:e1003424. 10.1371/journal.pcbi.1003424 24453953 PMC3894152

[B34] Wang Y. Eddy J. A. Price N. D. (2012). Reconstruction of genome-scale metabolic models for 126 human tissues using mcadre. BMC Syst. Biol. 6:153. 10.1186/1752-0509-6-153 23234303 PMC3576361

[B35] Yizhak K. Gaude E. Le Dévédec S. Waldman Y. Y. Stein G. Y. van de Water B.. (2014). Phenotype-based cell-specific metabolic modeling reveals metabolic liabilities of cancer. Elife 3:e03641. 10.7554/eLife.03641 25415239 PMC4238051

[B36] Zilliox M. J. Irizarry R. A. (2007). A gene expression bar code for microarray data. Nat. Methods 4, 911–913. 10.1038/nmeth1102 17906632 PMC3154617

[B37] Zur H. Ruppin E. Shlomi T. (2010). imat: an integrative metabolic analysis tool. Bioinformatics 26, 3140–3142. 10.1093/bioinformatics/btq602 21081510

