# Supplementary material for: Benchmarking Procedures for High-Throughput Context Specific Reconstruction Algorithms
Source: Front Physiol. 2016 Jan 22;6:410. doi: 10.3389/fphys.2015.00410 (PMC4722106; doi:10.3389/fphys.2015.00410)
Supplement: Supplementary File 1 — Supplementary tables and figures. Modifications of Recon to allow the reconstruction of hepatonet. Example of free NADPH production in hepatonet. [file SupplementaryFile1.pdf]

Supplementary Material: Benchmarking procedures for high-throughput context specific reconstruction algorithms

Maria Pires Pacheco [1], Thomas Pfau [1,2] and Thomas Sauter [1,*]

*Correspondence:
Thomas Sauter
thomas.sauter@uni.lu

1 SUPPLEMENTARY TABLES AND FIGURES

Table 1. Number, percentage of gene-associated reactions and percentage of reactions of each context-specific reconstruction that have a high, medium and low confidence score to be expressed at the protein level. An enrichment in high and medium confidence level is observed for discretization-based algorithms (GIMME, iMAT, FASTCORE z-score, FASTCORMICS and FASTCORMICS medium constrained.

algorithms	description	high	medium	low	not detected
	number of reactions	628	641	65	265
Recon	% of the reactions of the model	11 %	11 %	1 %	5 %
	% of the gene-associated reactions	17 %	17 %	2 %	7 %
	number of reactions	213	266	47	108
HepatoNet	% of the reactions of the model	9 %	11 %	2 %	5 %
	% of the gene-associated reactions	12 %	15 %	3 %	6 %
	number of reactions	518	444	47	126
GIMME	% of the reactions of the model	15 %	13 %	1 %	4 %
	% of the gene-associated reactions	25 %	21 %	2 %	6 %
	number of reactions	574	525	55	153
iMAT	% of the reactions of the model	16 %	14 %	2 %	4 %
	% of the gene-associated reactions	24 %	22 %	2 %	6 %
	number of reactions	453	499	55	155
iNIT	% of the reactions of the model	12 %	13 %	1 %	4 %
	% of the gene-associated reactions	16 %	18 %	2 %	6 %
	number of reactions	376	418	41	186
RegrEX	% of the reactions of the model	12 %	13 %	1 %	6 %
	% of the gene-associated reactions	15 %	16 %	2 %	7 %
	number of reactions	624	637	64	260
Akesson08	% of the reactions of the model	11 %	11 %	1 %	5 %
	% of the gene-associated reactions	17 %	17 %	2 %	7 %
	number of reactions	584	413	21	123
FASTCORE z-score	% of the reactions of the model	20 %	14 %	1 %	4 %
	% of the gene-associated reactions	28 %	20 %	1 %	6 %
	number of reactions	570	391	15	73
FASTCORMICS	% of the reactions of the model	21 %	15 %	1 %	3 %
	% of the gene-associated reactions	30 %	21 %	1 %	4 %
	number of reactions	481	343	15	66
FASTCORMICS	% of the reactions of the model	22 %	16 %	1 %	3 %
medium constrained	% of the gene-associated reactions	29 %	21 %	1 %	4 %

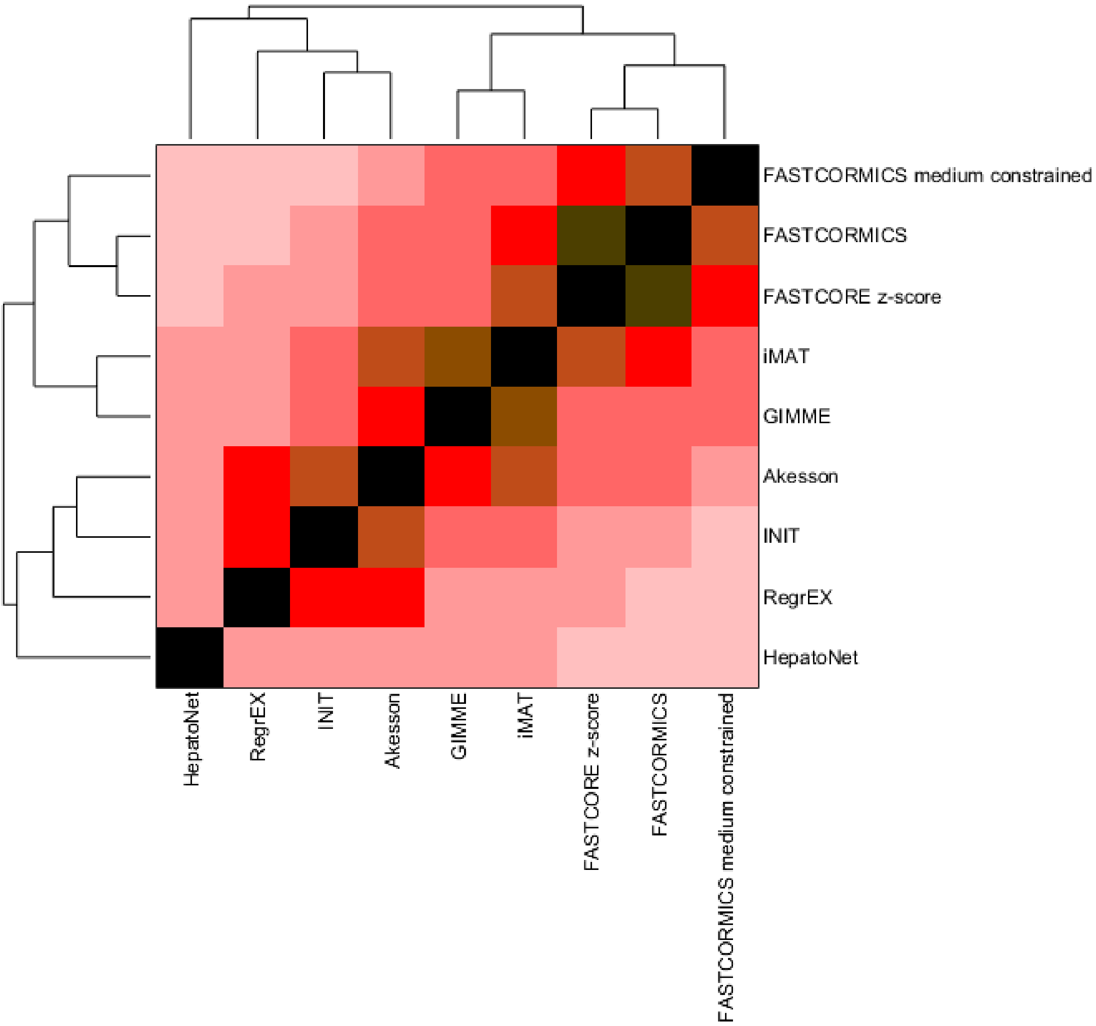

Figure 1. Similarity index of the models built by the different algorithms. The Jaccard index was computed for each pair of models, the rows and column were then clustered in function of the Euclidean distance. Contrary to what was expected, the output models of the tested algorithms, despite having been fed with the same input show a huge variability. The descritization-based algorithms (GIMME, iMAT, Akesson, FASTCORE, FASTCORMICS and FASTCORMICS no medium constrained) show the highest similarity levels.

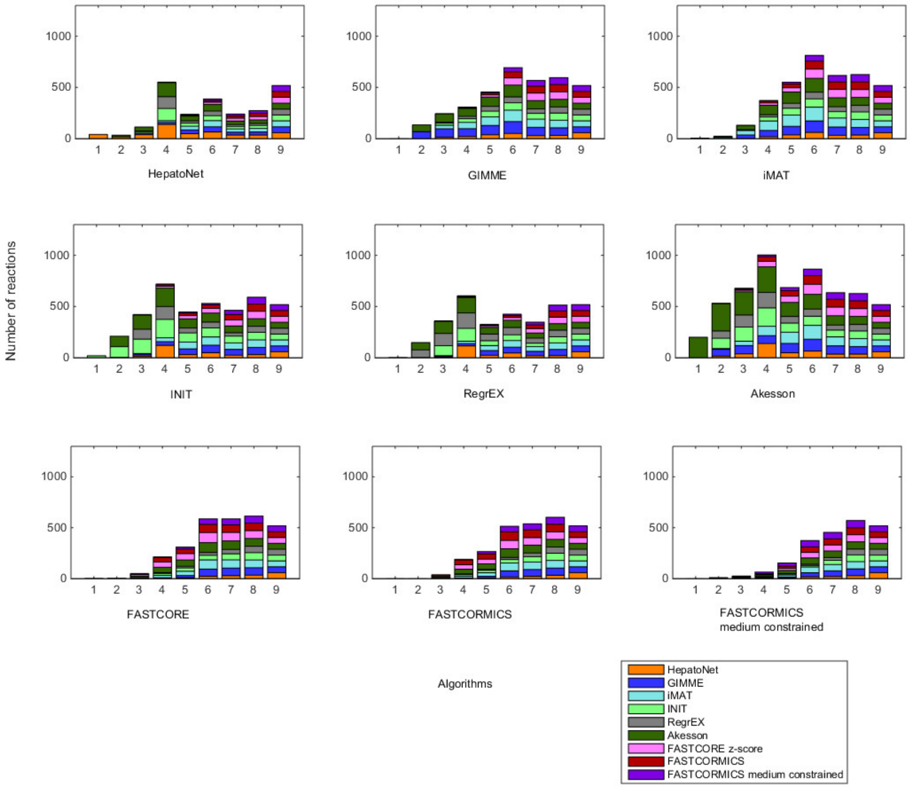

Figure 2. Reactions overlap: Each subplot represents HepatoNet or a model built by one of the tested algorithm. From left to right, in the top: HepatoNet, GIMME, iMAT, in the second row: INIT, RegrEx, Akesson in the third row: FASTCORE z-score, and FASTCORMICS no medium constraint. The different bars of the stacked boxplot illustrate the number of reactions that are common to 1, 2, 3, 4, 5 to all the models. The colour in the stacked plots represent the reactions of different models (HepatoNet (orange), the GIMME (dark blue), iMAT (light blue), INIT (green), RegrEx (gray), Akesson (dark green), FASTCORE z-score (pink), FASTCORMICS (brown) and FASTCORMICS no medium constrained (violet). The size of the colors areas are proportional to the number of reactions shared between the methods. A greater percentage of reactions in the GIMME, IMAT, FASTCORE, FASTCORMICS and FASTCORMICS medium constrained models are supported by at least 3 other algorithms.

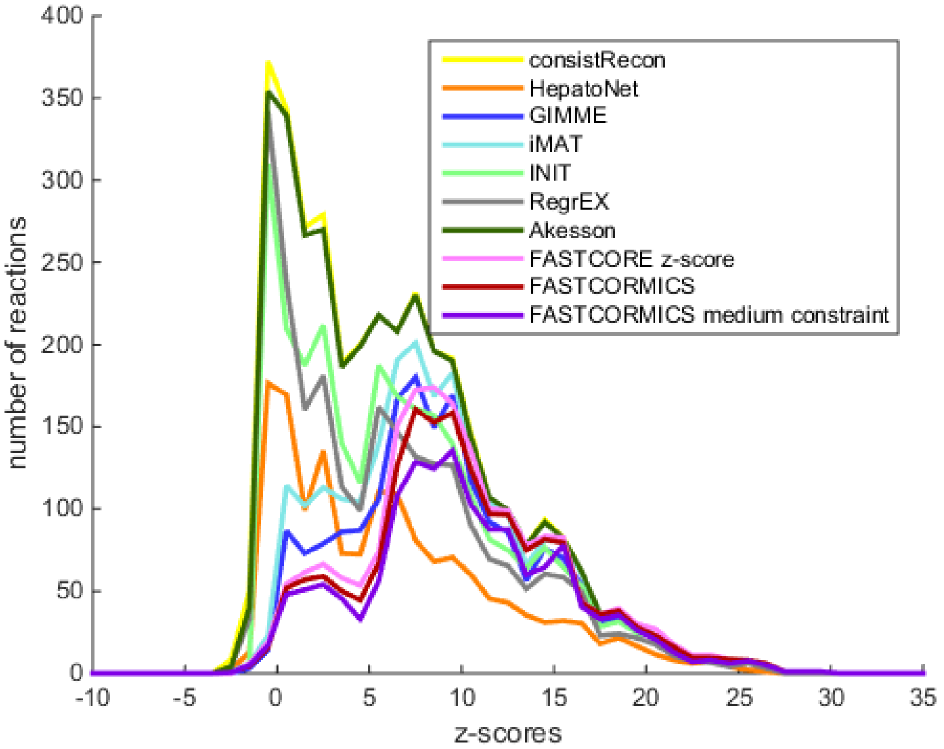

Figure 3. Confidence score at the transcriptomic level: Median z-score of the intensity measured in the liver samples to the median intensity distribution for the genes in an unexpressed context mapped the genes-associated reactions of Recon2 (yellow), HepatoNet (orange), the GIMME (dark blue), iMAT (light blue), INIT (green), RegrEx (gray), Akesson (dark green), FASTCORE z-score (pink), FASTCORMICS (brown) and FASTCORMICS no medium constrained (violet).
Discretization-based algorithms (GIMME, iMAT, FASTCORE, FASTCORMICS and FASTCORMICS medium constrained) are enriched for higher z-score values.

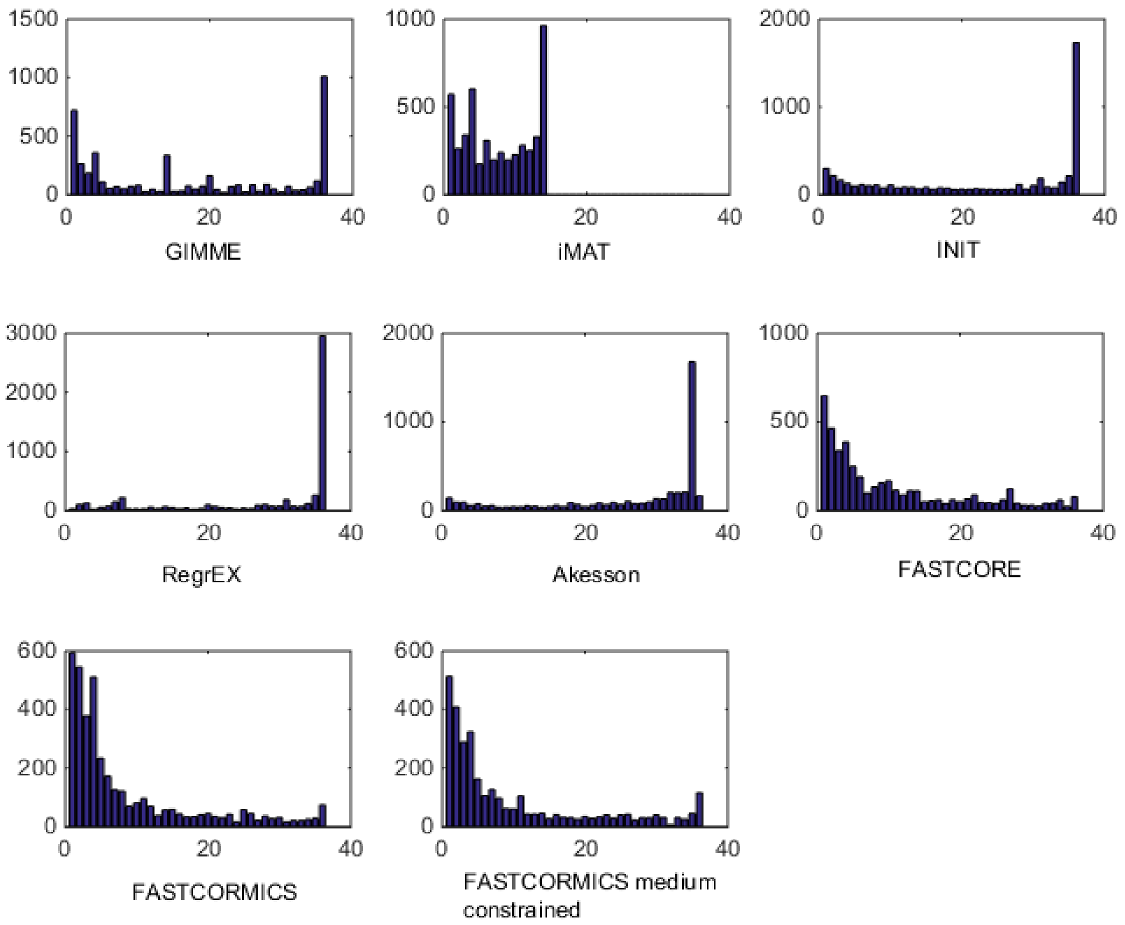

Figure 4.
Tissue specificity of reconstructed models. Number of reactions that are present in 1, 2, 3 up to 36 tissues models. For INIT and RegrEX, more than 1500 and 3000 reactions are present in all tissues models, while a similar number is present in all but one model created by the Akesson method. Due to computational complexity iMAT was only able to generate 14 out of 36 tissue models.

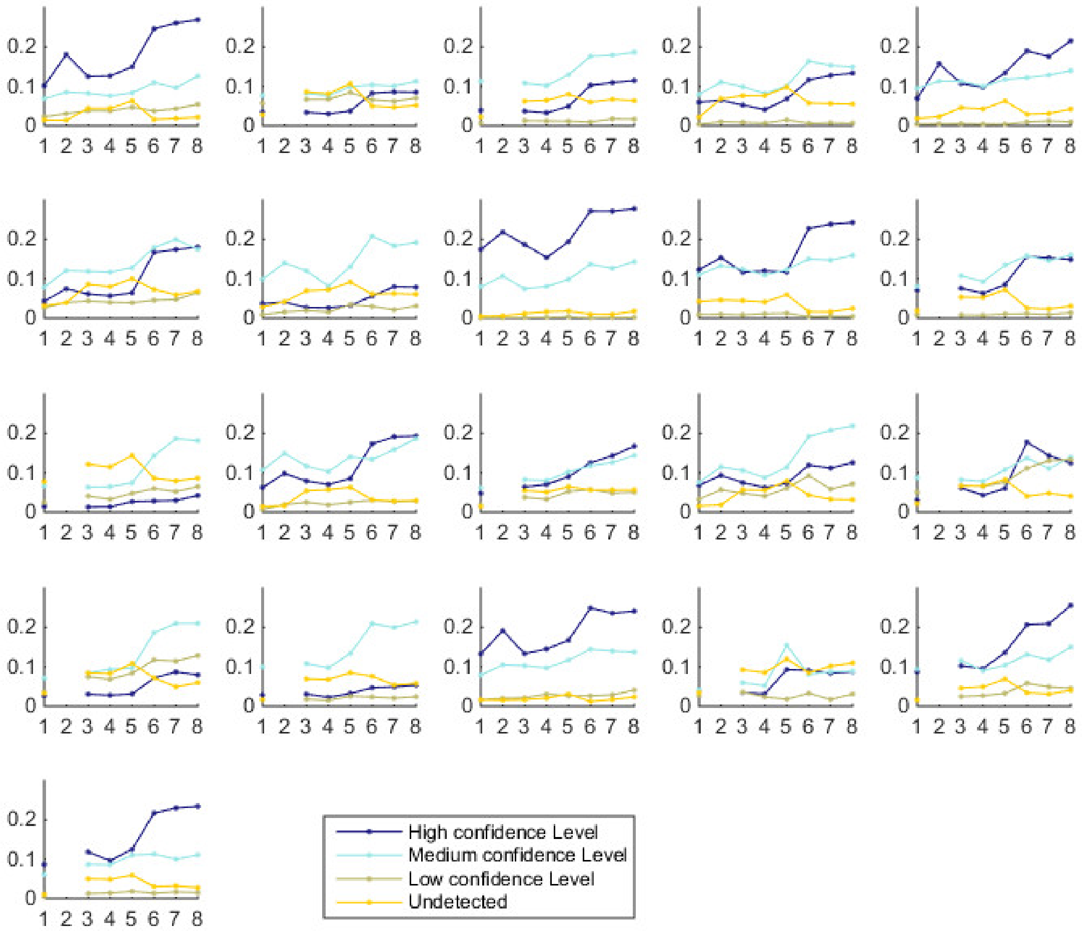

Figure 5. Percentage of reactions that are associated with high confidence (dark blue), medium confidence level (light blue), low confidence level (khaki) and not detected (yellow). Each subplot represent a different tissue. The x-axis represent the different algorithms: 1-GIMME, 2-iMAT, 3-INIT, 4-RegrEX, 5-Akesson, 6-FASTCORE z-score, 7-FASTCORMICS and 8-FASTCORMICS medium constrained and the y-axis the percentage of reactions.

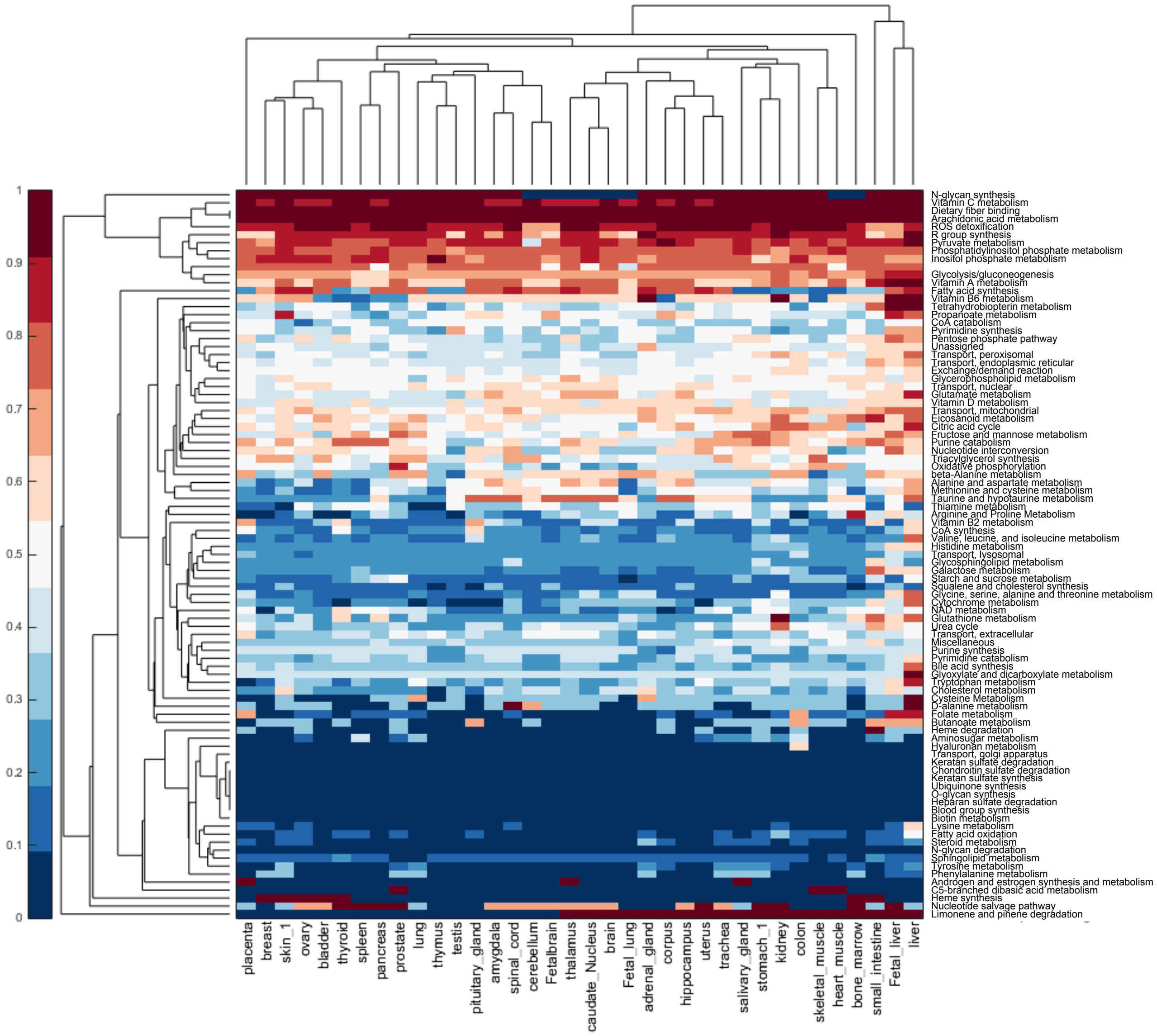
1
0.9
0.8
0.7
0.6
0.5
0.4
0.3
0.2
0.1
0
N-glycan synthesis
Vitamin C metabolism
Dietary fiber binding
Arachidonic acid metabolism
ROS detoxification
R group synthesis
Pyruvate metabolism
Phosphatidylinositol phosphate metabolism
Inositol phosphate metabolism
Glycolysis/gluconeogenesis
Vitamin A metabolism
Fatty acid synthesis
Vitamin B6 metabolism
Tetrahydrobiopterin metabolism
Propanoate metabolism
CoA catabolism
Pyrimidine synthesis
Pentose phosphate pathway
Unassigned
Transport, peroxisomal
Transport, endoplasmic reticular
Exchange/demand reaction
Glycerophospholipid metabolism
Transport, nuclear
Glutamate metabolism
Vitamin D metabolism
Transport, mitochondrial
Eicosanoid metabolism
Citric acid cycle
Fructose and mannose metabolism
Purine catabolism
Nucleotide interconversion
Triacylglycerol synthesis
Oxidative phosphorylation
beta-Alanine metabolism
Alanine and aspartate metabolism
Methionine and cysteine metabolism
Taurine and hypotaurine metabolism
Thiamine metabolism
Arginine and Proline Metabolism
Vitamin B2 metabolism
CoA synthesis
Valine, leucine, and isoleucine metabolism
Histidine metabolism
Transport, lysosomal
Glycosphingolipid metabolism
Galactose metabolism
Starch and sucrose metabolism
Squalene and cholesterol synthesis
Glycine, serine, alanine and threonine metabolism
Cytochrome metabolism
NAD metabolism
Glutathione metabolism
Urea cycle
Transport, extracellular
Miscellaneous
Purine synthesis
Pyrimidine catabolism
Bile acid synthesis
Glyoxylate and dicarboxylate metabolism
Tryptophan metabolism
Cholesterol metabolism
Cysteine Metabolism
D-alanine metabolism
Folate metabolism
Butanoate metabolism
Heme degradation
Aminosugar metabolism
Hyaluronan metabolism
Transport, golgi apparatus
Keratan sulfate degradation
Chondroitin sulfate degradation
Keratan sulfate synthesis
Ubiquinone synthesis
O-glycan synthesis
Heparan sulfate degradation
Blood group synthesis
Biotin metabolism
Lysine metabolism
Fatty acid oxidation
Steroid metabolism
N-glycan degradation
Sphingolipid metabolism
Tyrosine metabolism
Phenylalanine metabolism
Androgen and estrogen synthesis and metabolism
C5-branched dibasic acid metabolism
Heme synthesis
Nucleotide salvage pathway
Limonene and pinene degradation
placenta
breast
skin_1
ovary
bladder
thyroid
spleen
pancreas
prostate
lung
thymus
testis
pituitary_gland
amygdala
spinal_cord
cerebellum
Fetalbrain
thalamus
caudate_Nucleus
brain
Fetal_lung
adrenal_gland
corpus
hippocampus
uterus
trachea
salivary_gland
stomach_1
kidney
colon
skeletal_muscle
heart_muscle
bone_marrow
small_intestine
Fetal_liver
liver

GIMME

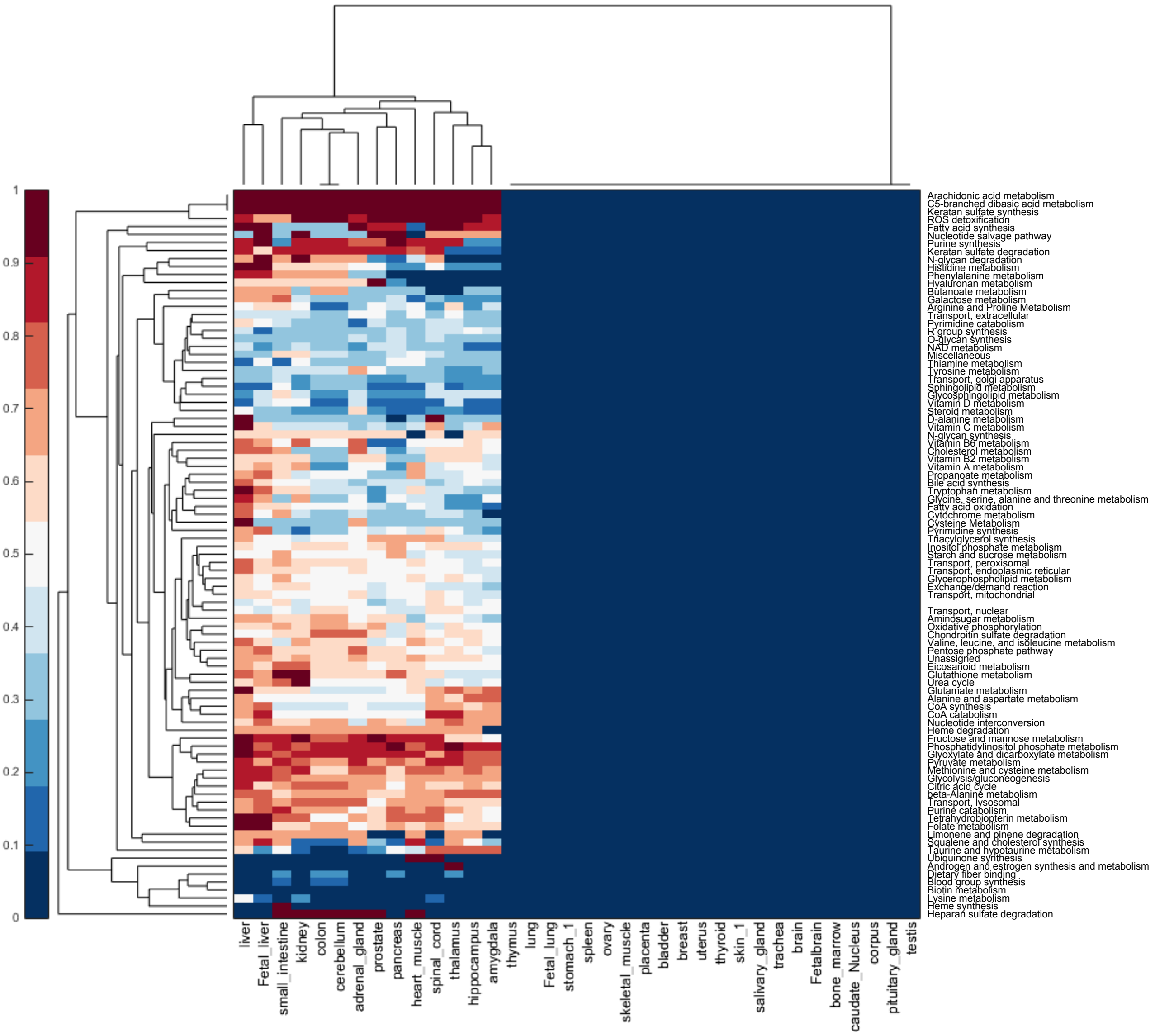
1
0.9
0.8
0.7
0.6
0.5
0.4
0.3
0.2
0.1
0
Arachidonic acid metabolism
C5-branched dibasic acid metabolism
Keratan sulfate synthesis
ROS detoxification
Fatty acid synthesis
Nucleotide salvage pathway
Purine synthesis
Keratan sulfate degradation
N-glycan degradation
Histidine metabolism
Phenylalanine metabolism
Hyaluronan metabolism
Butanoate metabolism
Galactose metabolism
Arginine and Proline Metabolism
Transport, extracellular
Pyrimidine catabolism
R group synthesis
O-glycan synthesis
NAD metabolism
Miscellaneous
Thiamine metabolism
Tyrosine metabolism
Transport, golgi apparatus
Sphingolipid metabolism
Glycosphingolipid metabolism
Vitamin D metabolism
Steroid metabolism
D-alanine metabolism
Vitamin C metabolism
N-glycan synthesis
Vitamin B6 metabolism
Cholesterol metabolism
Vitamin B2 metabolism
Vitamin A metabolism
Propanoate metabolism
Bile acid synthesis
Tryptophan metabolism
Glycine, serine, alanine and threonine metabolism
Fatty acid oxidation
Cytochrome metabolism
Cysteine Metabolism
Pyrimidine synthesis
Triacylglycerol synthesis
Inositol phosphate metabolism
Starch and sucrose metabolism
Transport, peroxisomal
Transport, endoplasmic reticular
Glycerophospholipid metabolism
Exchange/demand reaction
Transport, mitochondrial
Transport, nuclear
Aminosugar metabolism
Oxidative phosphorylation
Chondroitin sulfate degradation
Valine, leucine, and isoleucine metabolism
Pentose phosphate pathway
Unassigned
Eicosanoid metabolism
Glutathione metabolism
Urea cycle
Glutamate metabolism
Alanine and aspartate metabolism
CoA synthesis
CoA catabolism
Nucleotide interconversion
Heme degradation
Fructose and mannose metabolism
Phosphatidylinositol phosphate metabolism
Glyoxylate and dicarboxylate metabolism
Pyruvate metabolism
Methionine and cysteine metabolism
Glycolysis/gluconeogenesis
Citric acid cycle
beta-Alanine metabolism
Transport, lysosomal
Purine catabolism
Tetrahydrobiopterin metabolism
Folate metabolism
Limonene and pinene degradation
Squalene and cholesterol synthesis
Taurine and hypotaurine metabolism
Ubiquinone synthesis
Androgen and estrogen synthesis and metabolism
Dietary fiber binding
Blood group synthesis
Biotin metabolism
Lysine metabolism
Heme synthesis
Heparan sulfate degradation
liver
Fetal_liver
small_intestine
kidney
colon
cerebellum
adrenal_gland
prostate
pancreas
heart_muscle
spinal_cord
thalamus
hippocampus
amygdala
thymus
lung
Fetal_lung
stomach_1
spleen
ovary
skeletal_muscle
placenta
bladder
breast
uterus
thyroid
skin_1
salivary_gland
trachea
brain
Fetalbrain
bone_marrow
caudate_Nucleus
corpus
pituitary_gland
testis

iMAT

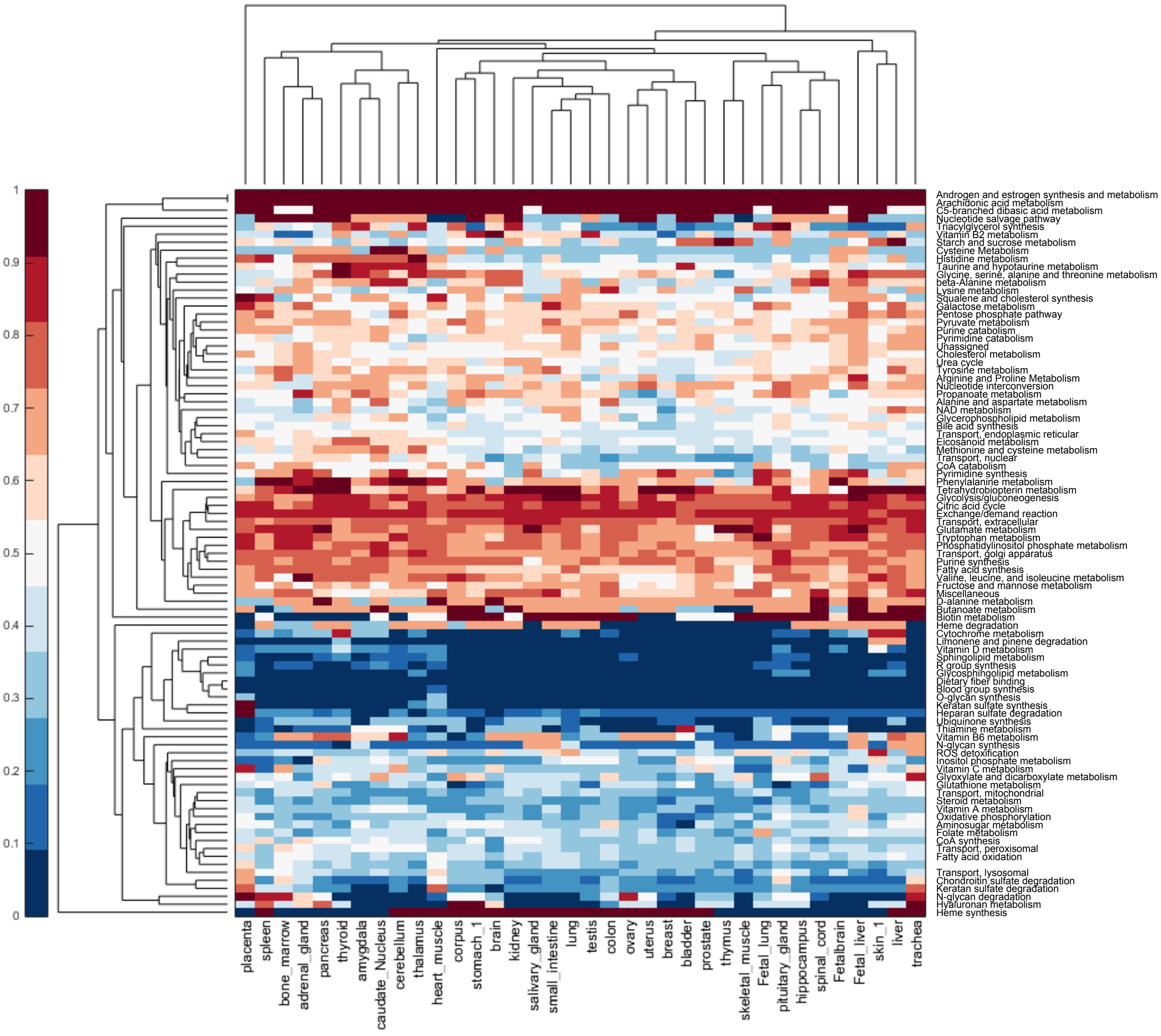
1
0.9
0.8
0.7
0.6
0.5
0.4
0.3
0.2
0.1
0
Androgen and estrogen synthesis and metabolism
Arachidonic acid metabolism
C5-branched dibasic acid metabolism
Nucleotide salvage pathway
Triacylglycerol synthesis
Vitamin B2 metabolism
Starch and sucrose metabolism
Cysteine Metabolism
Histidine metabolism
Taurine and hypotaurine metabolism
Glycine, serine, alanine and threonine metabolism
beta-Alanine metabolism
Lysine metabolism
Squalene and cholesterol synthesis
Galactose metabolism
Pentose phosphate pathway
Pyruvate metabolism
Purine catabolism
Pyrimidine catabolism
Unassigned
Cholesterol metabolism
Urea cycle
Tyrosine metabolism
Arginine and Proline Metabolism
Nucleotide interconversion
Propanoate metabolism
Alanine and aspartate metabolism
NAD metabolism
Glycerophospholipid metabolism
Bile acid synthesis
Transport, endoplasmic reticular
Eicosanoid metabolism
Methionine and cysteine metabolism
Transport, nuclear
CoA catabolism
Pyrimidine synthesis
Phenylalanine metabolism
Tetrahydrobiopterin metabolism
Glycolysis/gluconeogenesis
Citric acid cycle
Exchange/demand reaction
Transport, extracellular
Glutamate metabolism
Tryptophan metabolism
Phosphatidylinositol phosphate metabolism
Transport, golgi apparatus
Purine synthesis
Fatty acid synthesis
Valine, leucine, and isoleucine metabolism
Fructose and mannose metabolism
Miscellaneous
D-alanine metabolism
Butanoate metabolism
Biotin metabolism
Heme degradation
Cytochrome metabolism
Limonene and pinene degradation
Vitamin D metabolism
Sphingolipid metabolism
R group synthesis
Glycosphingolipid metabolism
Dietary fiber binding
Blood group synthesis
O-glycan synthesis
Keratan sulfate synthesis
Heparan sulfate degradation
Ubiquinone synthesis
Thiamine metabolism
Vitamin B6 metabolism
N-glycan synthesis
ROS detoxification
Inositol phosphate metabolism
Vitamin C metabolism
Glyoxylate and dicarboxylate metabolism
Glutathione metabolism
Transport, mitochondrial
Steroid metabolism
Vitamin A metabolism
Oxidative phosphorylation
Aminosugar metabolism
Folate metabolism
CoA synthesis
Transport, peroxisomal
Fatty acid oxidation
Transport, lysosomal
Chondroitin sulfate degradation
Keratan sulfate degradation
N-glycan degradation
Hyaluronan metabolism
Heme synthesis
placenta
spleen
bone_marrow
adrenal_gland
pancreas
thyroid
amygdala
caudate_Nucleus
cerebellum
thalamus
heart_muscle
corpus
stomach_1
brain
kidney
salivary_gland
small_intestine
lung
testis
colon
ovary
uterus
breast
bladder
prostate
thymus
skeletal_muscle
Fetal_lung
pituitary_gland
hippocampus
spinal_cord
Fetalbrain
Fetal_liver
skin_1
liver
trachea

INIT

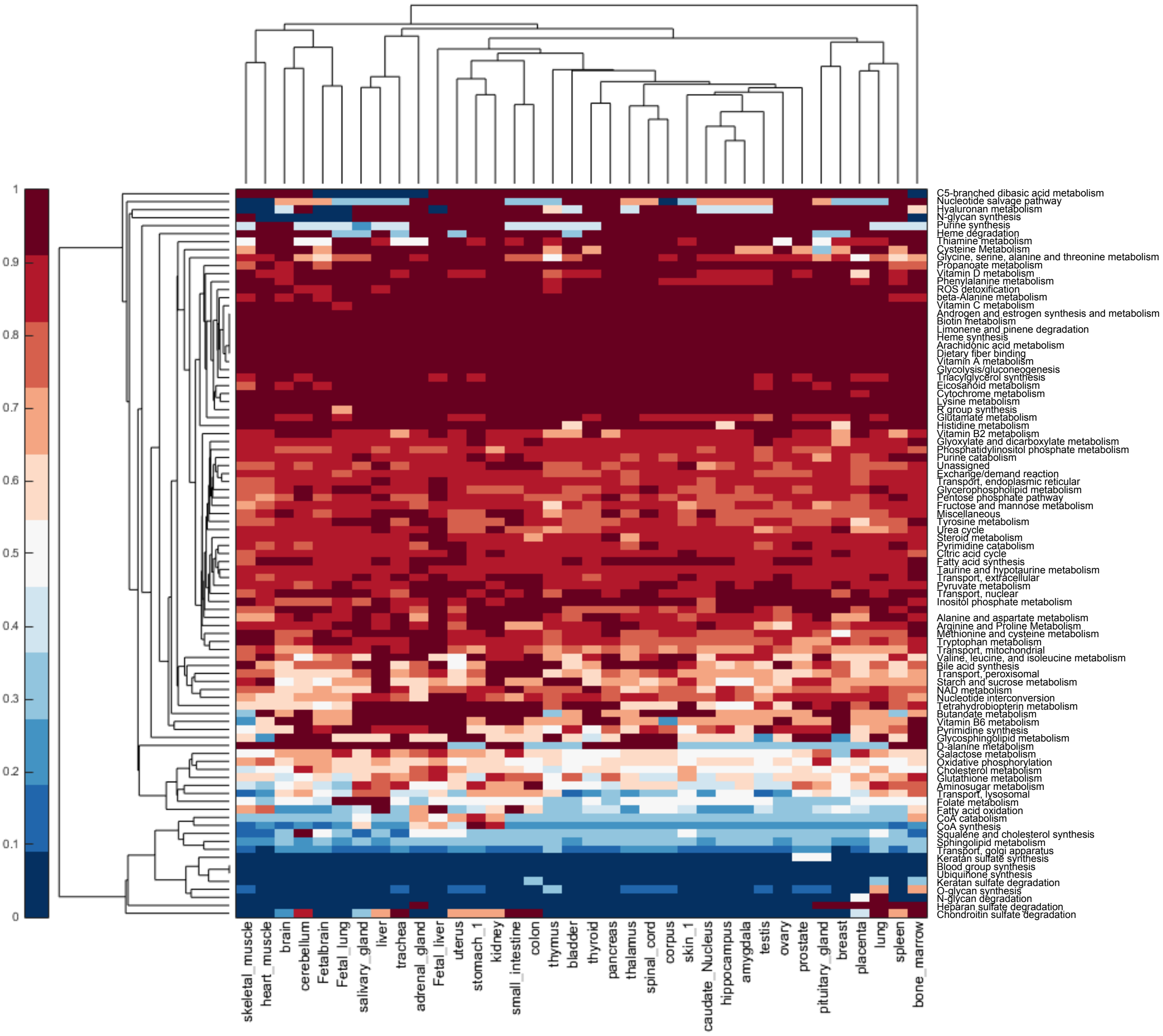
1
0.9
0.8
0.7
0.6
0.5
0.4
0.3
0.2
0.1
0
C5-branched dibasic acid metabolism
Nucleotide salvage pathway
Hyaluronan metabolism
N-glycan synthesis
Purine synthesis
Heme degradation
Thiamine metabolism
Cysteine Metabolism
Glycine, serine, alanine and threonine metabolism
Propanoate metabolism
Vitamin D metabolism
Phenylalanine metabolism
ROS detoxification
beta-Alanine metabolism
Vitamin C metabolism
Androgen and estrogen synthesis and metabolism
Biotin metabolism
Limonene and pinene degradation
Heme synthesis
Arachidonic acid metabolism
Dietary fiber binding
Vitamin A metabolism
Glycolysis/gluconeogenesis
Triacylglycerol synthesis
Eicosanoid metabolism
Cytochrome metabolism
Lysine metabolism
R group synthesis
Glutamate metabolism
Histidine metabolism
Vitamin B2 metabolism
Glyoxylate and dicarboxylate metabolism
Phosphatidylinositol phosphate metabolism
Purine catabolism
Unassigned
Exchange/demand reaction
Transport, endoplasmic reticular
Glycerophospholipid metabolism
Pentose phosphate pathway
Fructose and mannose metabolism
Miscellaneous
Tyrosine metabolism
Urea cycle
Steroid metabolism
Pyrimidine catabolism
Citric acid cycle
Fatty acid synthesis
Taurine and hypotaurine metabolism
Transport, extracellular
Pyruvate metabolism
Transport, nuclear
Inositol phosphate metabolism
Alanine and aspartate metabolism
Arginine and Proline Metabolism
Methionine and cysteine metabolism
Tryptophan metabolism
Transport, mitochondrial
Valine, leucine, and isoleucine metabolism
Bile acid synthesis
Transport, peroxisomal
Starch and sucrose metabolism
NAD metabolism
Nucleotide interconversion
Tetrahydrobiopterin metabolism
Butanoate metabolism
Vitamin B6 metabolism
Pyrimidine synthesis
Glycosphingolipid metabolism
D-alanine metabolism
Galactose metabolism
Oxidative phosphorylation
Cholesterol metabolism
Glutathione metabolism
Aminosugar metabolism
Transport, lysosomal
Folate metabolism
Fatty acid oxidation
CoA catabolism
CoA synthesis
Squalene and cholesterol synthesis
Sphingolipid metabolism
Transport, golgi apparatus
Keratan sulfate synthesis
Blood group synthesis
Ubiquinone synthesis
Keratan sulfate degradation
O-glycan synthesis
N-glycan degradation
Heparan sulfate degradation
Chondroitin sulfate degradation
skeletal_muscle
heart_muscle
brain
cerebellum
Fetalbrain
Fetal_lung
salivary_gland
liver
trachea
adrenal_gland
Fetal_liver
uterus
stomach_1
kidney
small_intestine
colon
thymus
bladder
thyroid
pancreas
thalamus
spinal_cord
corpus
skin_1
caudate_Nucleus
hippocampus
amygdala
testis
ovary
prostate
pituitary_gland
breast
placenta
lung
spleen
bone_marrow

Akesson

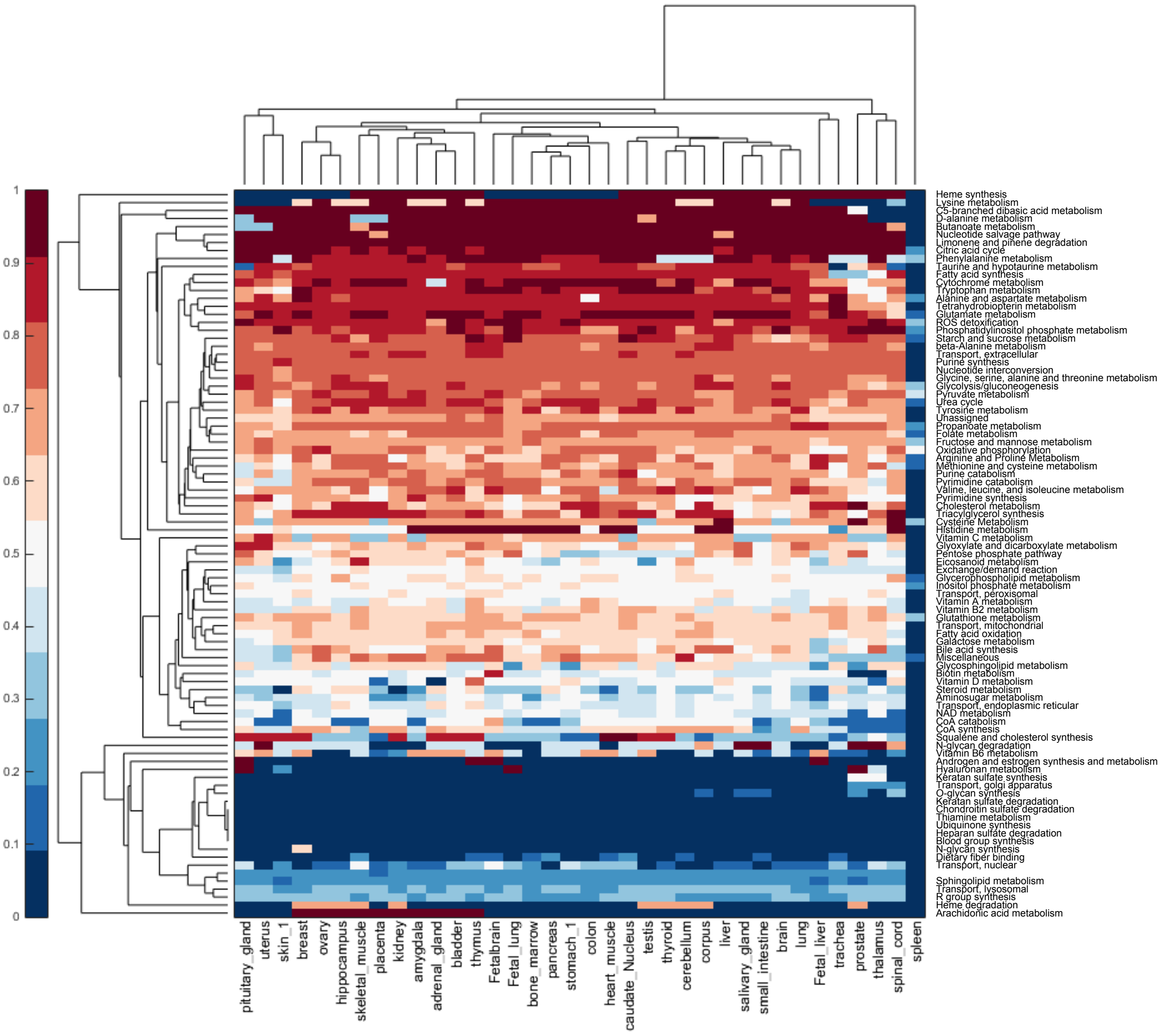
1
0.9
0.8
0.7
0.6
0.5
0.4
0.3
0.2
0.1
0
Heme synthesis
Lysine metabolism
C5-branched dibasic acid metabolism
D-alanine metabolism
Butanoate metabolism
Nucleotide salvage pathway
Limonene and pinene degradation
Citric acid cycle
Phenylalanine metabolism
Taurine and hypotaurine metabolism
Fatty acid synthesis
Cytochrome metabolism
Tryptophan metabolism
Alanine and aspartate metabolism
Tetrahydrobiopterin metabolism
Glutamate metabolism
ROS detoxification
Phosphatidylinositol phosphate metabolism
Starch and sucrose metabolism
beta-Alanine metabolism
Transport, extracellular
Purine synthesis
Nucleotide interconversion
Glycine, serine, alanine and threonine metabolism
Glycolysis/gluconeogenesis
Pyruvate metabolism
Urea cycle
Tyrosine metabolism
Unassigned
Propanoate metabolism
Folate metabolism
Fructose and mannose metabolism
Oxidative phosphorylation
Arginine and Proline Metabolism
Methionine and cysteine metabolism
Purine catabolism
Pyrimidine catabolism
Valine, leucine, and isoleucine metabolism
Pyrimidine synthesis
Cholesterol metabolism
Triacylglycerol synthesis
Cysteine Metabolism
Histidine metabolism
Vitamin C metabolism
Glyoxylate and dicarboxylate metabolism
Pentose phosphate pathway
Eicosanoid metabolism
Exchange/demand reaction
Glycerophospholipid metabolism
Inositol phosphate metabolism
Transport, peroxisomal
Vitamin A metabolism
Vitamin B2 metabolism
Glutathione metabolism
Transport, mitochondrial
Fatty acid oxidation
Galactose metabolism
Bile acid synthesis
Miscellaneous
Glycosphingolipid metabolism
Biotin metabolism
Vitamin D metabolism
Steroid metabolism
Aminosugar metabolism
Transport, endoplasmic reticular
NAD metabolism
CoA catabolism
CoA synthesis
Squalene and cholesterol synthesis
N-glycan degradation
Vitamin B6 metabolism
Androgen and estrogen synthesis and metabolism
Hyaluronan metabolism
Keratan sulfate synthesis
Transport, golgi apparatus
O-glycan synthesis
Keratan sulfate degradation
Chondroitin sulfate degradation
Thiamine metabolism
Ubiquinone synthesis
Heparan sulfate degradation
Blood group synthesis
N-glycan synthesis
Dietary fiber binding
Transport, nuclear
Sphingolipid metabolism
Transport, lysosomal
R group synthesis
Heme degradation
Arachidonic acid metabolism
pituitary_gland
uterus
skin_1
breast
ovary
hippocampus
skeletal_muscle
placenta
kidney
amygdala
adrenal_gland
bladder
thymus
Fetalbrain
Fetal_lung
bone_marrow
pancreas
stomach_1
colon
heart_muscle
caudate_Nucleus
testis
thyroid
cerebellum
corpus
liver
salivary_gland
small_intestine
brain
lung
Fetal_liver
trachea
prostate
thalamus
spinal_cord
spleen

RegrEX

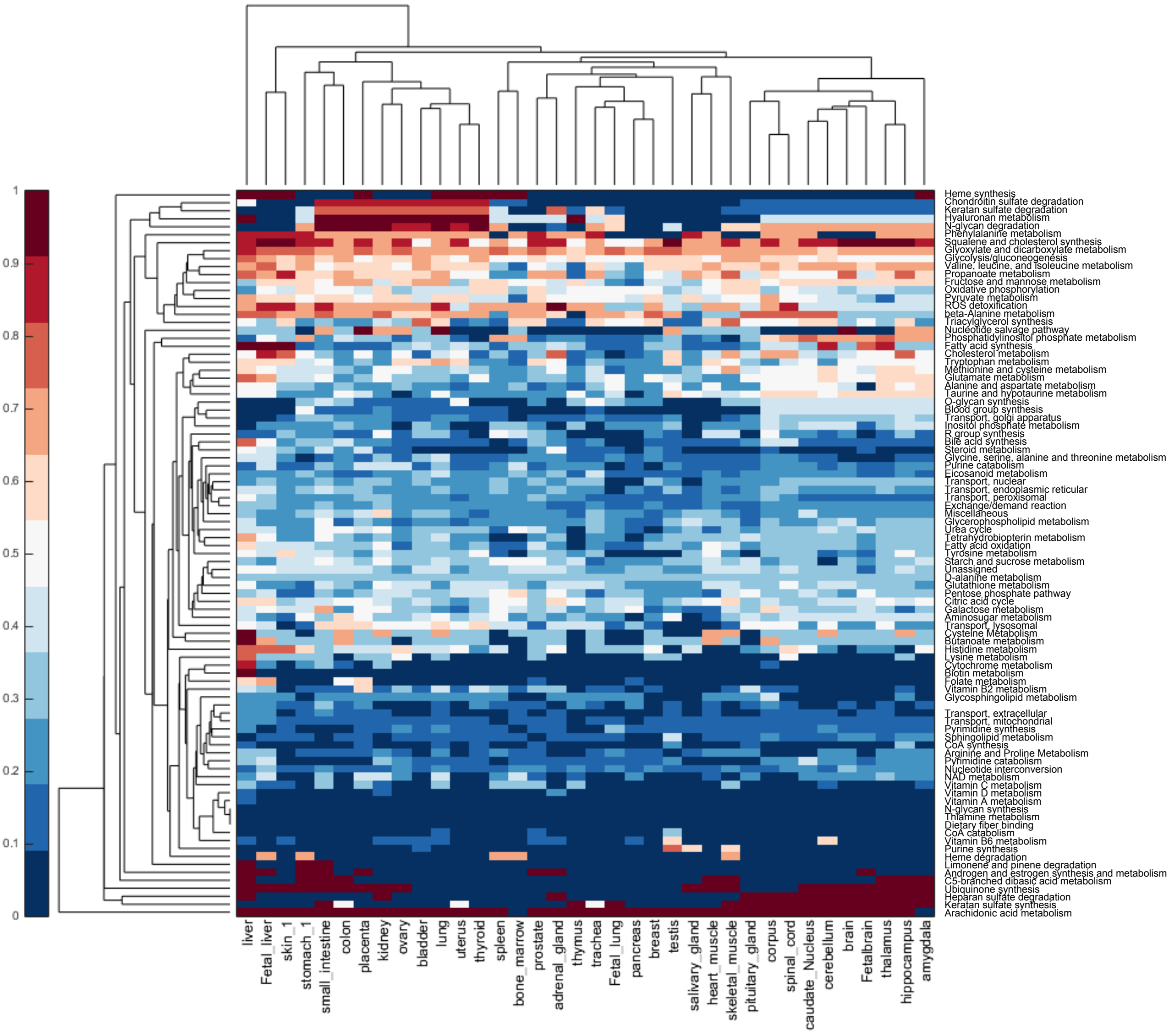
1
0.9
0.8
0.7
0.6
0.5
0.4
0.3
0.2
0.1
0
Heme synthesis
Chondroitin sulfate degradation
Keratan sulfate degradation
Hyaluronan metabolism
N-glycan degradation
Phenylalanine metabolism
Squalene and cholesterol synthesis
Glyoxylate and dicarboxylate metabolism
Glycolysis/gluconeogenesis
Valine, leucine, and isoleucine metabolism
Propanoate metabolism
Fructose and mannose metabolism
Oxidative phosphorylation
Pyruvate metabolism
ROS detoxification
beta-Alanine metabolism
Triacylglycerol synthesis
Nucleotide salvage pathway
Phosphatidylinositol phosphate metabolism
Fatty acid synthesis
Cholesterol metabolism
Tryptophan metabolism
Methionine and cysteine metabolism
Glutamate metabolism
Alanine and aspartate metabolism
Taurine and hypotaurine metabolism
O-glycan synthesis
Blood group synthesis
Transport, golgi apparatus
Inositol phosphate metabolism
R group synthesis
Bile acid synthesis
Steroid metabolism
Glycine, serine, alanine and threonine metabolism
Purine catabolism
Eicosanoid metabolism
Transport, nuclear
Transport, endoplasmic reticular
Transport, peroxisomal
Exchange/demand reaction
Miscellaneous
Glycerophospholipid metabolism
Urea cycle
Tetrahydrobiopterin metabolism
Fatty acid oxidation
Tyrosine metabolism
Starch and sucrose metabolism
Unassigned
D-alanine metabolism
Glutathione metabolism
Pentose phosphate pathway
Citric acid cycle
Galactose metabolism
Aminosugar metabolism
Transport, lysosomal
Cysteine Metabolism
Butanoate metabolism
Histidine metabolism
Lysine metabolism
Cytochrome metabolism
Biotin metabolism
Folate metabolism
Vitamin B2 metabolism
Glycosphingolipid metabolism
Transport, extracellular
Transport, mitochondrial
Pyrimidine synthesis
Sphingolipid metabolism
CoA synthesis
Arginine and Proline Metabolism
Pyrimidine catabolism
Nucleotide interconversion
NAD metabolism
Vitamin C metabolism
Vitamin D metabolism
Vitamin A metabolism
N-glycan synthesis
Thiamine metabolism
Dietary fiber binding
CoA catabolism
Vitamin B6 metabolism
Purine synthesis
Heme degradation
Limonene and pinene degradation
Androgen and estrogen synthesis and metabolism
C5-branched dibasic acid metabolism
Ubiquinone synthesis
Heparan sulfate degradation
Keratan sulfate synthesis
Arachidonic acid metabolism
liver
Fetal_liver
skin_1
stomach_1
small_intestine
colon
placenta
kidney
ovary
bladder
lung
uterus
thyroid
spleen
bone_marrow
prostate
adrenal_gland
thymus
trachea
Fetal_lung
pancreas
breast
testis
salivary_gland
heart_muscle
skeletal_muscle
pituitary_gland
corpus
spinal_cord
caudate_Nucleus
cerebellum
brain
Fetalbrain
thalamus
hippocampus
amygdala

FASTCORE

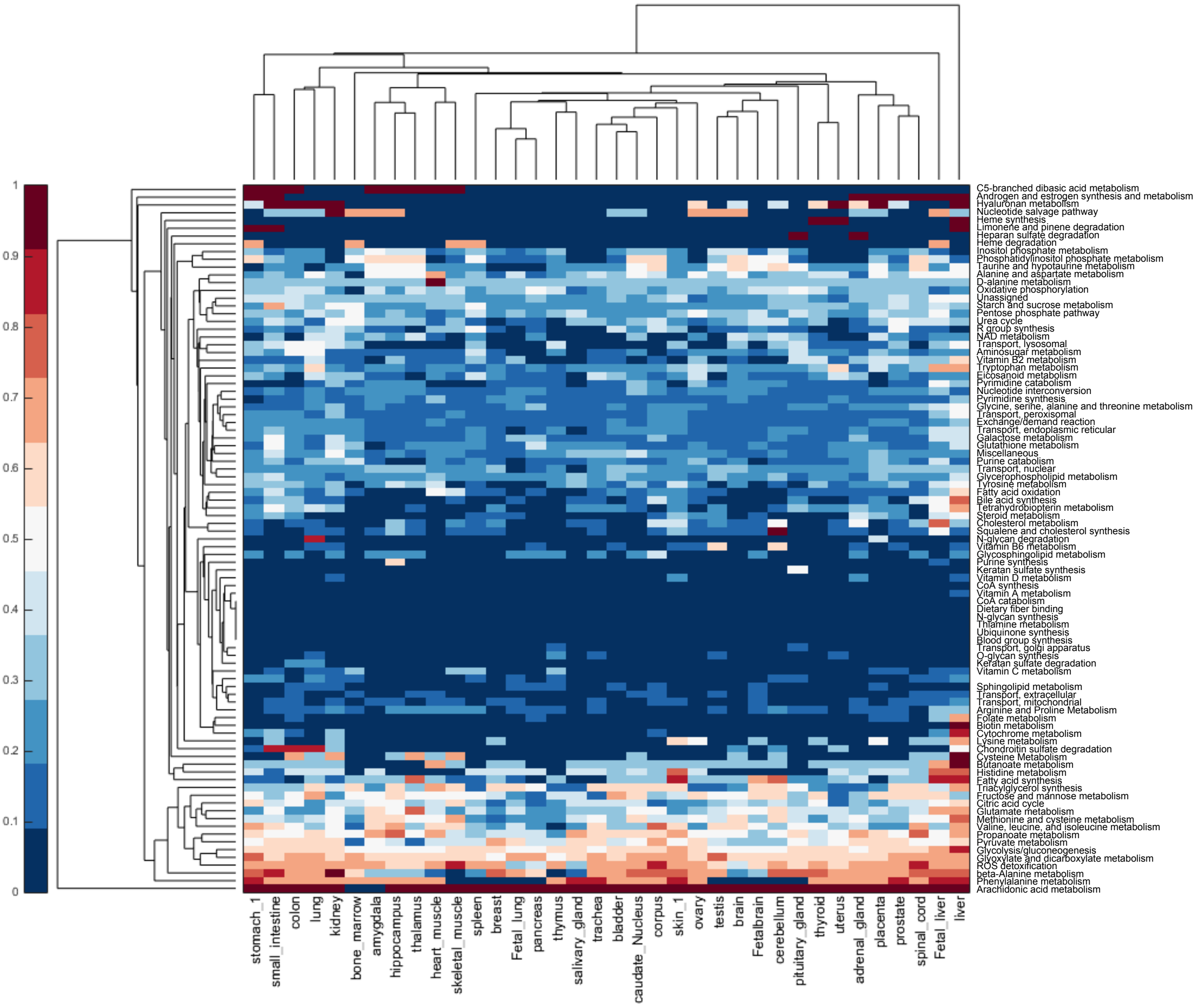

FASTCORMICS

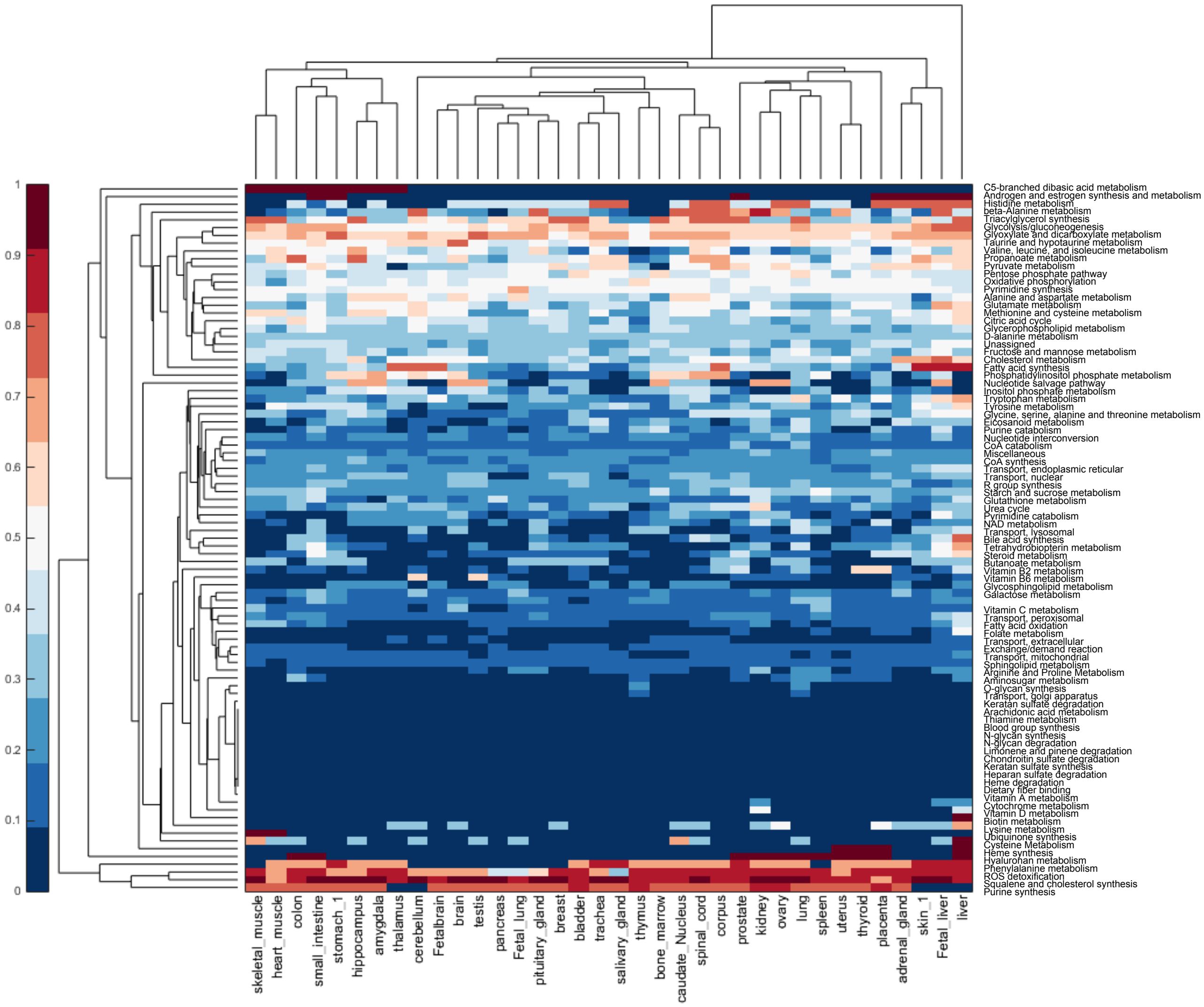

FASTCORMICS no medium constraint

Figure 6: Cluster plots of the tissue models built by the different tested algorithms: The fraction of active reactions in each pathway as defined by Recon2 was computed for each tissue model. The models were then cluster in function of the Euclidean distance.

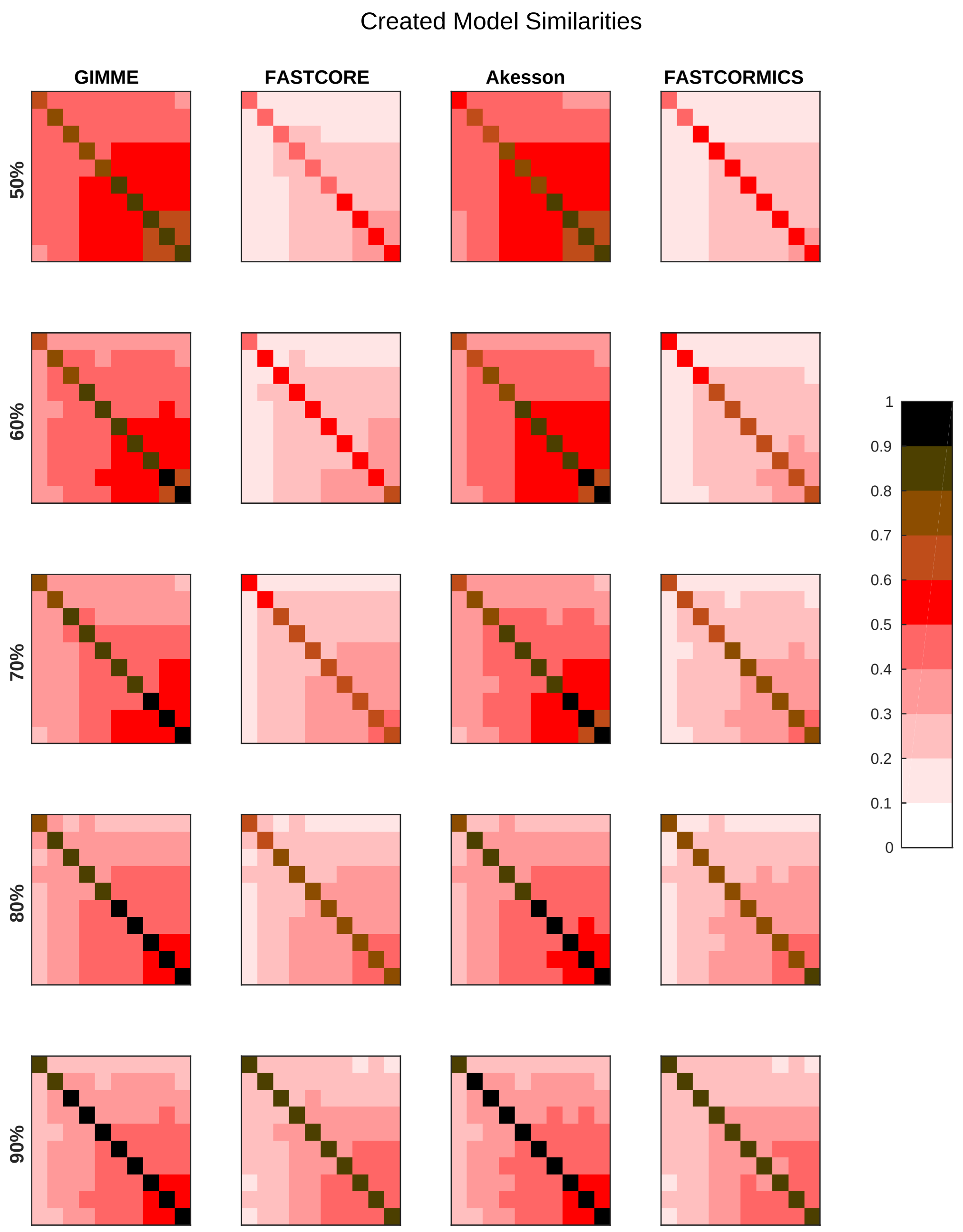

Figure 7. Resolution power: The plot shows the mean Jaccard distance between the networks generated by the different algorithms along with the distances of the original models. Each square represents the comparison between all reconstructions of one model and all reconstructions of another model, with the diagonal representing the comparison of all reconstructed model of the same model. The diagonal can therefore be an indicator for robustness (the brighter, the more similar the models) while the off diagonal indicates similarities between the generated models and is therefore an indicator for specificity to the input (the darker, the more distinct the generated models). When 90% of the data is available, all the algorithms are able to distinguish variations between the different models. But with a less complete data set, inclusive algorithms (here GIMME and Akesson) lose in specificity. It would also be expected that when only 50% of the data is available, the robustness decreases. But GIMME and Akesson only show a modest decrease of robustness.

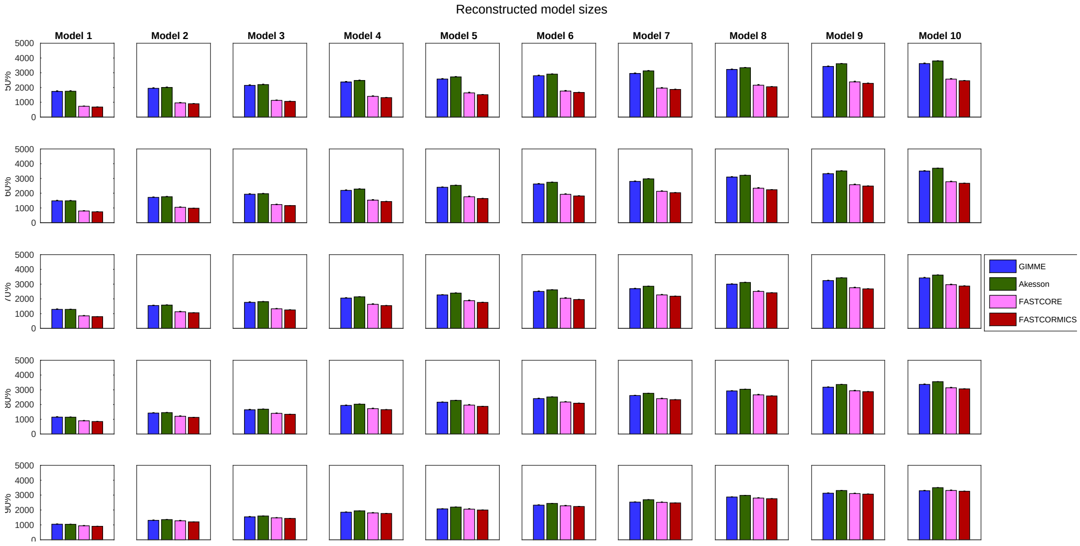

Figure 8. Model Sizes of the reconstructed Models. The target models had the following sizes: Model 1: 961, Model2 : 1276, Model 3: 1528, Model 4: 1876, Model 5: 2123, Model 6: 2377, Model 7:2629, Model 8: 2935, Model 9: 3264, Model 10: 3455 The plots show that for a more complete input set, all algorithms tend to approach the real model size.

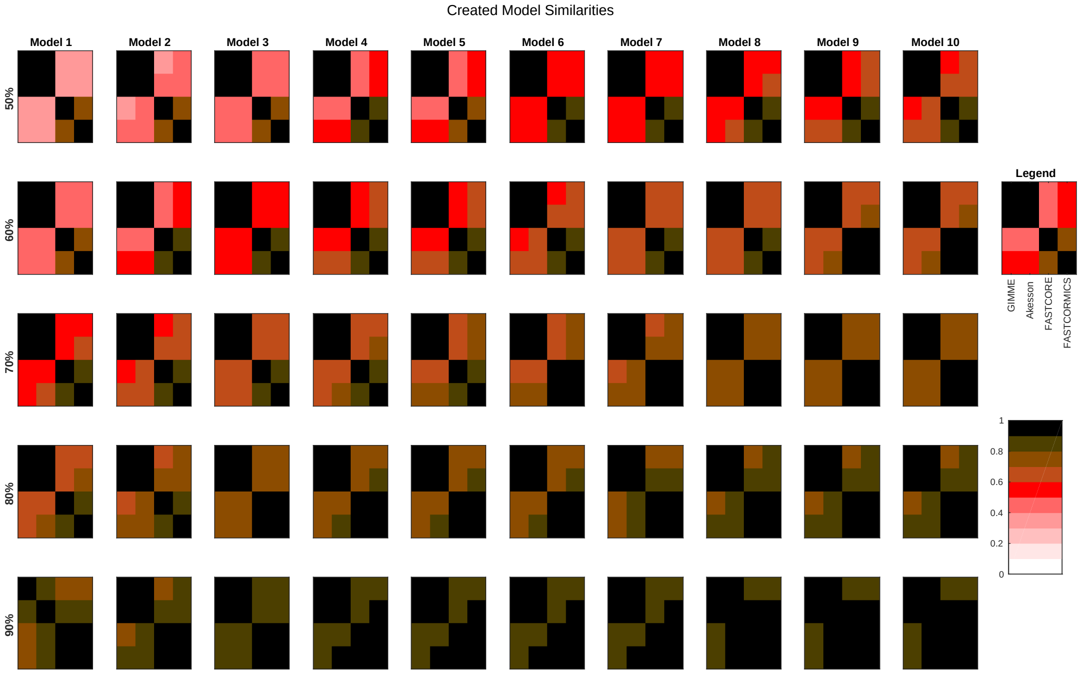

Figure 9. The plots show the mean Jaccard distance between the networks generated by the different algorithms for several artificial models and input percentages. For each algorithm, the corresponding networks (using the same input data) are compared. Smaller models (e.g. Model 1) tend to yield more distinguishable results, while larger models (due to a larger fraction of common reactions), tend to yield more similar networks. Overall, the difference between inclusive (GIMME/Akesson) and exclusive (Fastcore/FASTCORMICS) algorithms is clearly visible.

2 MODIFICATIONS FOR RECON TO ALLOW THE RECONSTRUCTION OF HEPATONET

- A reaction to create tag_hs from 3 stearoyl-CoAs was introduced to allow synthesis of biomass without an uptake of unidentified fatty acids.
- HepatoNet metabolites were matched to Recon Metabolites
- 2 reactions converting NH3 into NH4 were removed.
- 2 reactions converting Carbonate into HCO3.
- Recon reactions were matched to HepatoNet Reactions
- Reversibilities of HepatoNet were used for all Recon reactions that were mapped.
- Uptake Exchange reactions were adjusted to a maximal uptake of 1 (since there are several reactions which can take up Proteins and those would lead to a biomass above the upper bound.
- Gene AI971036 was converted to 3417 (according to GeneCards)
- In addition, the following reversibilities were adjusted:
- GTHRDt was made irreversible (active transport of glutathione into the mitochondrion can otherwise generate ATP in Recon2).
- C16txc was made irreversible , as it led to the production of free ATP in Recon2
- GTHPm was made irreversible as it allowed the free generation of reductants in Recon2.
- BILDGLCURte was made irreversible , as it led to the production of free ATP in Recon2
- The GPRs used in Recon2 version 4 were used as those are more consistent.

Our HepatoNet was than constructed by extracting all reactions which are in HepatoNet from the modified Recon network and adding all Importers and Exporters from Recon.

Reaction	Formula using Recon ids	Flux
$r0083$	$HC01434[m] <=> co2[m] + akg[m]$	1
$r0400$	$nad[c] + o2[c] + acnam[c] <=> h2o[c] + nadh[c] + HC01115[c]$	0.5
$r0425$	$nad[m] + icit[m] <=> nadh[m] + HC01434[m]$	1
$r0617$	$nadp[c] + 4hpro_LT[c] <=> nadph[c] + 1p3h5c[c]$	0.5
$r0668$	$ctp[c] + HC01115[c] <=> ppi[c] + HC01162[c]$	0.5
$r1459$	$ppi[c] <=> ppi[n]$	0.5
$r1461$	$cmpacna[c] <=> cmpacna[n]$	0.5
$r0082$	$HC01434[c] <=> co2[c] + akg[c]$	-1
$r0268$	$nadph[c] + o2[c] + cmpacna[c] <=> h2o[c] + nadp[c] + HC01162[c]$	-0.5
$r0269$	$ctp[n] + acnam[n] <=> ppi[n] + cmpacna[n]$	-0.5
$r0422$	$nadp[c] + icit[c] <=> nadph[c] + HC01434[c]$	-1
$r0615$	$nad[c] + 4hpro_LT[c] <=> nadh[c] + 1p3h5c[c]$	-0.5
$r0868$	$co2[c] <=> co2[m]$	-1
$r1147$	$akg[c] + icit[m] <=> akg[m] + icit[c]$	-1
$r1460$	$ctp[c] <=> ctp[n]$	-0.5
$r1462$	$acnam[c] <=> acnam[n]$	-0.5

Table 2. Overview of methods used for validation of automated tissue specific reconstruction algorithms.

3 EXAMPLE OF FREE NADPH PRODUCTION IN HEPATONET

An example solution can be found in Table 2

The Flux distribution can be split into the following reactions:
r0083 + r0425 - r0082 - r0422 - r0868 - r1147 :
NAD(m) + NADPH(c) <=> NADH(m) + NADP(c) (1)

r1459 - r1462 - r1460 - r0269 :
cmpacna[c] + ppi[c] <=> ctp[c] + acnam[c] (2)

r0615 - r0615 :
NADH(c) + NADP(c) <=> NADPH(c) + NAD(c) (3)

r0400 + r0668 - r0268 :
NAD[c] + acnam[c]+ NADP[c] + ctp[c] <=> cmpacna[c] + ppi[c] + NADPH[c] + NADH[c] (4)

With (4) + (2) clearly producing free reductant.
